# Synovial monocytes contribute to chronic inflammation in childhood-onset arthritis via IL-6/STAT signalling and cell-cell interactions

**DOI:** 10.3389/fimmu.2023.1190018

**Published:** 2023-05-22

**Authors:** Tobias Schmidt, Alma Dahlberg, Elisabet Berthold, Petra Król, Sabine Arve-Butler, Emilia Rydén, Seyed Morteza Najibi, Anki Mossberg, Anders A. Bengtsson, Fredrik Kahn, Bengt Månsson, Robin Kahn

**Affiliations:** ^1^ Department of Pediatrics, Clinical Sciences Lund, Lund University, Lund, Sweden; ^2^ Wallenberg Center for Molecular Medicine, Lund University, Lund, Sweden; ^3^ Department of Rheumatology, Clinical Sciences Lund, Lund University, Lund, Sweden; ^4^ Department of Clinical Sciences, Division of Infection Medicine, Lund University, Lund, Sweden

**Keywords:** monocyte, inflammation, juvenile idiopathic arthritis – JIA, synovial fluid (SF), IL-6, rheumatology

## Abstract

**Introduction:**

Monocytes are key effector cells in inflammatory processes. We and others have previously shown that synovial monocytes in childhood-onset arthritis are activated. However, very little is known about how they contribute to disease and attain their pathological features. Therefore, we set out to investigate the functional alterations of synovial monocytes in childhood-onset arthritis, how they acquire this phenotype, and whether these mechanisms could be used to tailorize treatment.

**Methods:**

The function of synovial monocytes was analysed by assays believed to reflect key pathological events, such as T-cell activation-, efferocytosis- and cytokine production assays using flow cytometry in untreated oligoarticular juvenile idiopathic arthritis (oJIA) patients (n=33). The effect of synovial fluid on healthy monocytes was investigated through mass spectrometry and functional assays. To characterize pathways induced by synovial fluid, we utilized broad-spectrum phosphorylation assays and flow cytometry, as well as inhibitors to block specific pathways. Additional effects on monocytes were studied through co-cultures with fibroblast-like synoviocytes or migration in transwell systems.

**Results:**

Synovial monocytes display functional alterations with inflammatory and regulatory features, e.g., increased ability to induce T-cell activation, resistance to cytokine production following activation with LPS and increased efferocytosis. *In vitro*, synovial fluid from patients induced the regulatory features in healthy monocytes, such as resistance to cytokine production and increased efferocytosis. IL-6/JAK/STAT signalling was identified as the main pathway induced by synovial fluid, which also was responsible for a majority of the induced features. The magnitude of synovial IL-6 driven activation in monocytes was reflected in circulating cytokine levels, reflecting two groups of low *vs.* high local and systemic inflammation. Remaining features, such as an increased ability to induce T-cell activation and markers of antigen presentation, could be induced by cell-cell interactions, specifically *via* co-culture with fibroblast-like synoviocytes.

**Conclusions:**

Synovial monocytes in childhood-onset arthritis are functionally affected and contribute to chronic inflammation, e.g., *via* promoting adaptive immune responses. These data support a role of monocytes in the pathogenesis of oJIA and highlight a group of patients more likely to benefit from targeting the IL-6/JAK/STAT axis to restore synovial homeostasis.

## 1 Introduction

Monocytes are key players in the innate immune system, with roles in antigen presentation, cytokine production and phagocytosis. They are crucial in the defence against invading organisms, but can be detrimental in autoimmune diseases, such as arthritis. They are found in the synovial fluid of patients with adult arthritis, displaying several markers of activation compared to circulating monocytes, such as CD16, HLA-DR and toll-like receptors (TLRs) (1, 2). Furthermore, synovial monocytes and macrophages display a state of polarization (3). Polarization, or activation, is the process in which these cells respond to the environment and acquire distinct phenotypes, a process that also influences the cells’ effector functions (4). Traditionally, these are classified as pro-inflammatory or anti-inflammatory/regulatory phenotypes, although polarization most likely represents a continuum were these cells display features from both endpoints. Multiple cytokines found within the inflamed joint can induce polarization, which mainly signals through the JAK/STAT pathway (3, 4). Interestingly, polarization may differ between different forms of arthritis, which may have implications in the function of these cells in different arthritides (5, 6). Synovial monocytes and macrophages are believed to drive pro-inflammatory processes *via* cell-cell interactions, e.g., with fibroblast-like synoviocytes and T-cells, and production of soluble mediators such as cytokines (7, 8). In addition, features of regulatory monocytes and macrophages, such as angiogenesis, can facilitate chronic inflammation in arthritis (9, 10). CD163, a classic marker of regulatory monocytes and macrophages, correlate with inflammation in spondyloarthritis (11). Finally, these cells can also be potent sources of inflammatory cytokines (12). Thus, both inflammatory and regulatory polarization may be detrimental in chronic inflammation.

Chronic childhood-onset arthritis (juvenile idiopathic arthritis, JIA) is the most common rheumatic disease in children (13). JIA is a heterogenous disease that includes different subtypes, but the most common one in the western world is oligoarticular JIA (oJIA), which accounts for 30-60% of all patients (14, 15). The children usually present at preschool age, are antinuclear-antibody (ANA) positive and have a high risk of developing uveitis (14). The pathogenesis of oJIA is largely unknown, but the interplay between innate and adaptive immunity is believed to play an important role. The affected joint is characterized by hyperplasia, inflammation, and presence of infiltrating immune cells from both the innate and adaptive immune system (16). T- and B-cells are implied in the disease course, given the genetic relationship to MHC class II alleles and the presence of ANAs (16–18). Though, the contribution of members from the innate immune system, such as monocytes, is becoming increasingly recognized (19–22). However, in contrast to adult-onset rheumatoid arthritis, the environmental contribution to disease initiation is believed to be minor and thus childhood-onset JIA is a better model to study the underlying inflammatory response.

We and others have previously shown that synovial monocytes from patients with childhood-onset arthritis are polarized, with both inflammatory and regulatory features (19, 23). This sentence should be: "Monocytes and macrophages are believed to contribute to the pathogenesis, e.g., through cytokine production (24), but data on the role of monocytes as regulators in arthritis is lacking. Here, we set out to investigate how synovial monocytes are functionally affected and contribute to the pathogenesis, the mechanisms and pathways driving this phenotype, and the potential impact of these pathways in a clinical setting.

## 2 Methods

### 2.1 Patient material and clinical characteristics

Patients (n=33) with oligoarticular JIA (oJIA) according to the International League of Associations for Rheumatology (ILAR) criteria at the Department of Pediatric Rheumatology, Skåne University Hospital, Sweden between 2016-2022, were included in this study when undergoing therapeutic joint aspiration. This study was approved by the Regional Ethical Review Board for southern Sweden (2016/128). Patients were included upon written informed consent from the patients and/or their legal guardians. The patients had not received any disease-modifying anti-rheumatic drugs (DMARDs) or steroids, neither oral nor intra-articular, in the last 6 months prior to inclusion, but may have been administered non-steroid anti-inflammatory drugs (NSAIDs). The patient characteristics are described in **Table 1**. Synovial fluid (SF) and blood (in either EDTA-, serum-, or heparin tubes (BD Biosciences)) were collected.

**Table 1:** Overview of the patient cohort. Clinical and laboratory data of the 33 patients included in this study.

	Number of patients (%)	Data availability (%)
Oligoarthritis, persistent	28 (85)	33 (100)
Oligoarthritis, extended	5 (15)	33 (100)
Sex, female	25 (76)	33 (100)
NSAID	22 (67)	33 (100)
Uveitis	4 (12)	32 (97)
ANA-positive	25 (76)	33 (100)
Anti-CCP-positive	0 (0)	24 (73)
RF-positive	0 (0)	24 (73)
	Median (IQR)	Data availability (%)
^§^Disease duration, months	8 (84)	33 (100)
^#^Age, years	11 (6.5)	33 (100)
Active joint count	1 (1)	33 (100)
Blood WBC (10^6^/ml)	6.49 (2.03)	25 (76)
SF WBC (10^6^/ml)	4.01 (3.93)	26 (79)

^§^Disease duration at time of sampling. ^#^Age at time of diagnosis; ANA, Anti-nuclear antibodies; NSAID, Non-steroidal anti-inflammatory drugs; CCP, Cyclic citrullinated peptide; RF, Rheumatoid factor; WBC, White blood cells; SF, Synovial fluid.

### 2.2 Sample preparation

EDTA blood was used as control for analysis of surface markers (see below). For serum, the blood was allowed to clot for 1hr at room temperature followed by centrifugation at 1900g, 10min. Next, the serum was aliquoted and stored at -80°C until use. The heparin blood was used as a control for functional assays and monocyte isolation (described below).

A proportion of synovial fluid (SF) was used for functional studies and the rest was centrifuged at 500g, 10min. The SF was collected and centrifuged a second time at 800g, 10min to generate cell-free SF. Then, it was frozen at -80°C in aliquots until use. The remaining synovial cell fraction was washed once with PBS and subsequently resuspended in PBS with 0.5% BSA. Peripheral blood mononuclear cells (PBMCs), from heparinized blood, and synovial fluid mononuclear cells (SFMCs), from the synovial cell fraction, were isolated through density gradient centrifugation (Lymphoprep, Axis-Shield) at 620g, 20min with low break. The PBMCs and SFMCs were washed twice with PBS. Monocytes were further isolated from PBMCs and SFMCs using CD14^+^ magnetic bead separation (Miltenyi) as per the manufacturer’s instructions. The purified monocytes were counted and then used in subsequent assays described below.

### 2.3 Mesoscale cytokine measurement

CRP and SAA were measured in plasma (diluted 1/5000) using the vascular injury panel 2 (Mesoscale Diagnostics) according to the manufacturer’s instructions. IL-1, IL-6, IL-8 and TNF were analysed in plasma (diluted 1/2) and SF (diluted 1/25) using the pro-inflammatory panel 2 (4-plex). Finally, IFNα2a was measured in plasma and SF (both neat) using the S-PLEX human IFNα2a kit. Data was processed and concentrations were calculated using the Discovery Workbench software (Mesoscale, version 4.0).

### 2.4 Monocyte isolation and polarization *in vitro*

Monocytes were isolated from freshly isolated PBMCs, as described above, from healthy controls upon informed consent (n=10, median age 43, 50% female). Monocytes were next cultured overnight in 96-well plates (Falcon) at 1x10^6^cells/ml in RPMI-1640 medium with 2.05mM L-glutamine supplemented with either 20% patient serum or 20% paired SF to generate *in vitro* polarized monocytes. Each experimental section was performed using monocytes from at least two different donors unless otherwise stated. For blocking assays, monocytes were pre-incubated for 25min at 37°C with 1µM tofacitinib or 100ng/ml tocilizumab before the addition of SF. We confirmed that the drugs had minor effect on viability [**Supplementary Figure 1A**, and the concentrations of tofacitinib and tocilizumab used are in line with previous reports (25, 26)].

### 2.5 Surface marker analysis

For patients, synovial cells in PBS with 0.5% BSA at 2x10^6^cells/ml or 100µl of EDTA blood were incubated with two antibody mixes. First mix: anti-CD3 (clone UCHT-1, BV786, 1:50, BD), CD19 (clone HIB-19, Bv786, 1:125, BD), CD56 (clone NCAM 16.2, BV786, 1:125, BD), CD14 (clone 63D3, 1:50, Biolegend), CD66b (clone G10F5, alexa fluor 647, 1:50, BD), CD16 (clone 3G8, PerCP Cy5.5, 1:50, BD), MerTK (clone 590H11G1E3, PE, 1:100, Biolegend) and CD86 (clone FUN-1, BV650, 1:100, BD). The second mix contained the lineage antibodies (CD3, CD19, CD56, CD66b and CD14) and anti-HLA-DR, DP, DQ (clone Tü39, PerCP Cy5.5, 1:100, Biolegend) for 25min, RT. The cells were washed once with PBS before analysis using flow cytometry (CytoFLEX, Beckman Coulter). A subset of patients (n=5) was initially evaluated for differences in viability in CD14^+^ cells through live/dead staining (ThermoFisher) staining which were considered minor (**Supplementary Figure 1B**). Gating strategy for surface marker analysis can be found in **Supplementary Figure 2A**.

As a control to SF and patient serum, pooled normal human serum (NHS, Sigma-Aldrich) was included in the surface marker experiments. *In vitro* polarized monocytes were detached using ice-cold PBS/1mM EDTA and gentle pipetting. Next, they were washed with PBS and stained with anti-CD16, MerTK, CD86 and HLA, all diluted 1:200, for 25min, RT. Finally, they were washed once more with PBS and analysed by flow cytometry.

### 2.6 STAT phosphorylation

For patients, 100µl of heparinized blood or 100µl of whole SF in polypropylene FACS tubes (Falcon) were stained with (CD66b (clone G10F5, alexa fluor 700, 1:100, Biolegend), CD14 (clone HCD14, BV421, 1:100, Biolegend), CD3 (clone UCHT-1, BV510, 1:100, BD), CD19 (clone SJ25C1, BV510, 1:200, BD) and CD56 (NCAM16.2, BV510, 1:200, BD) in 100µl of PBS with 0.5% BSA. Simultaneously IFNγ (5ng/ml, R&D Systems), IL-4 (5ng/ml, R&D Systems), and IL-6 (5ng/ml, R&D Systems) were added to one set of tubes. Tubes not receiving cytokines and unstained tubes served as controls. The cells were incubated for 15min, 37°C. Next, 2ml of lyse/fix (BD) was added to each tube and incubated for another 10min, 37°C followed by centrifugation at 500g, 8min. The cells were then washed once with PBS and permeabilized as described below.

For *in vitro*, monocytes from healthy controls at 1x10^6^/ml in RPMI-1640 medium supplemented with 0.2% BSA were stimulated with 20% SF or 20% paired serum for 10min, 37°C to induce phosphorylation. In some experiments, monocytes were pre-incubated for 25min, 37°C with tofacitinib or tocilizumab (see above) before stimulation. Two TNF inhibitors, etanercept and infliximab, were tested as negative controls in n=3 donors activated with 20% SF from a pool of 8 SF donors (**Supplementary Figure 3**). Monocytes were subsequently fixated (CytoFix, BD) for 15min, 37°C before centrifugation.

Cells, either patient monocytes or *in vitro* polarized monocytes, were permeabilized (Perm Buffer III, BD) for 30min on ice followed by two washes with PBS. The patients’ monocytes were next stained for anti-STAT1-pY701 (clone 4a, alexa fluor 647, 1:100, BD), STAT3-pY705 (clone 4/P-STAT3, PE, 1:100, BD) and STAT6-pY641 (clone 18/p-Stat6, alexa fluor 488, 1:100, BD), whilst the *in vitro* polarized monocytes were stained with STAT1, STAT3 and NFkBp65-pS529 (clone K10-895, alexa fluor 488, 1:100, BD) in PBS supplemented with 0.5% BSA for 25min. Finally, the monocytes were washed once with PBS before analysis (CytoFLEX). Gating strategy for analysis of STAT phosphorylation in patients can be found in **Supplementary Figure 2B**.

### 2.7 T-cell isolation and proliferation

PBMCs were isolated from healthy controls using density centrifugation as described previously. CD4^+^ T-cells were isolated from the PBMC fraction using the EasySep™ CD4^+^ T-cell isolation kit (Stemcell Technologies) as per the manufacturer’s instructions. The T-cells were stained with 2µM CellTrace Violet (Invitrogen). A 96-well plate (Eppendorf) was coated with anti-CD3 (1:1000, Clone OKT3, Invitrogen) for 90min. The coating solution was removed prior to use. Wells without coating served as negative controls.

Monocytes from patients or *in vitro* polarization, as described above, were counted (XN-350, Sysmex) and resuspended in RPMI-1640 supplemented with 10% foetal calf serum, 2mM L-glutamine and PenStrep. Next, monocytes and T-cells at a 1:10 ratio (monocytes:T-cells) were added to the coated plate in a total volume of 200µl. The cells were incubated for 72h, 37°C, at 5% CO_2_. The cells were detached through gentle pipetting, centrifuged, and stained with anti-CD3 (clone UCHT1, alexa fluor 700, 1:200), anti-CD25 (clone M-A251, PerCP Cy5.51:200), anti-HLA-DR (clone G46-6, APC-H71:200) and anti-CTLA-4 (clone BNI3, PE, 1:50), all from BD, for 25min, RT. Finally, the cells were washed once with PBS and analysed using flow cytometry (CytoFLEX).

### 2.8 LPS activated cytokine production

For patients, heparinzed blood or fresh synovial fluid were diluted 1:1 with RPMI-1640 medium in polypropylene FACS tubes. Next, 0.5µl of golgiplug (BD) was added followed or not by 10ng/ml of LPS. The cells were incubated for 4hrs, 37°C, 5% CO_2_. For the last 15min, the cells were stained with anti-CD3 (clone UCHT-1, BV510, BD, 1:125), anti-CD19 (clone HIB19, BV786, BD, 1:150), CD14 (clone 63D3, alexa fluor (AF) 700, Biolegend, 1:125). Cells were fixated and lysed with BD lyse/fix solution for 10min, 37°C and subsequently washed two times with PBS. Permeabilization was performed by 10min incubation with 1ml of BD wash/perm (BD) followed by centrifugation and staining with anti-IL1ß (clone JK1B-1, AF647, Biolegend), IL-6 (cloneMQ2-13A5, PE/Cy7, Biolegend), IL-8 (clone E8N1, AF488, Biolegend) and TNF (cloneMAb11, BV650, BD) all diluted 1:50, for 25min, RT. The tubes were washed a final time with PBS before analysis using flow cytometry (CytoFLEX). Cells not receiving golgiplug or LPS were used to set the gates.

Healthy monocytes were resuspended in RPMI-1640 medium and seeded at 1x10^6^cells/ml with 20% serum or 20% paired SF in a 96-well plate. 0.5µl of golgiplug was added to each well followed by activation or not with 1ng/ml of LPS. The cells were incubated for 4hrs at 37°C, 5% CO_2_. Next, the cells were detached with PBS/1mM EDTA and gentle pipetting and washed once with PBS. They were subsequently fixated and permeabilized (CytoFix, BD) for 20min, 4°C. Thereafter, the monocytes were washed once with BD wash/perm and stained with the anti-IL-1ß, IL-6, IL-8 and TNF as above, all diluted 1:100 for 25min, RT. Finally, the cells were washed a final time and analysed by flow cytometry (CytoFLEX). Cells not receiving golgiplug or LPS were used to set the gates. Gating strategy can be found in **Supplementary Figure 2C**.

### 2.9 Efferocytosis

Neutrophils from heparinized blood of healthy donors were isolated through density centrifugation (Lymphoprep) as described above, followed by sedimentation of red blood cells for 20min using 1.5% Dextran T500 (Pharmacosmos) in saline. The remaining cells were transferred to a new tube and washed once with PBS. Residual red blood cells were lysed with sterile H_2_O for 25 seconds before restoration of isotonicity. 10^7^ neutrophils were resuspended in PBS and stained with 2µM of CellTrace Violet (CTV, Invitrogen) for 20min, 37°C. Extracellular dye was quenched by addition of RPMI-1640 medium supplemented with 10% FCS for 5min before centrifugation. The neutrophils were resuspended in serum-poor medium [RPMI-1640 medium supplemented with 1% normal human serum (Sigma-Aldrich)] at 5x10^6^ cells/ml and cultured for 24hrs at 37°C, 5% CO_2_ to induce apoptosis. Next day, the neutrophils were filtered through a cell-strainer cap (Falcon), centrifuged, and resuspended at 1x10^6^ cells/ml in RPMI-1640 supplemented with 10% of the neutrophil donor’s serum. Apoptosis was confirmed for each experiment through Annexin V staining, and monocytes were defined as CD14^+^CD66b^-^ to exclude bound, but not internalized, neutrophils (**Supplementary Figure 4A**).

For *in vitro* polarized monocytes (see above), medium was replaced with medium containing 1x10^5^ neutrophils, and the plate was incubated for 3hrs, 37°C, 5% CO_2_. For patients, 1x10^5^ freshly isolated monocytes, from either blood or SF, were resuspended in medium containing 1x10^5^ neutrophils, plated, and incubated as above. The cells were detached with cold PBS/1mM EDTA and gentle pipetting. Finally, the cells were washed once with PBS and stained with anti-CD14 (clone 63D3, Alexa Fluor 700, 1:200, Biolegend) and anti-CD66b (clone G10F5, FITC, 1:50, BD) for 25min, RT, followed by a final wash before analysis by flow cytometry (CytoFLEX). Monocytes were defined as CD14^+^CD66b^-^ to exclude monocytes that bound, but didn’t internalize, neutrophils (**Supplementary Figure 4B**). Percentage of CTV^+^ monocytes were used for analysis, and monocytes not receiving neutrophils were used to set the gates.

### 2.10 Phagocytosis

Phagocytosis was assessed using a bead-based phagocytosis assay (Cayman). Briefly, *in vitro* polarized monocytes’ medium was replaced with RPMI-1640 supplemented with 10% of the monocyte donor’s serum and diluted FITC-labelled opsonized beads (final dilution: 1/500). Binding of the beads, rather than phagocytosis, to the monocytes was assessed through incubation on ice (data not shown). Phagocytosis was performed for 30min, 37°C. Cells were detached with ice-cold PBS/1mM EDTA and surface bound beads were quenched with a 2min incubation with trypan blue on ice. Cells were subsequently washed twice with PBS before analysis by flow cytometry (CytoFLEX). The gates were set using cells that did not receive beads.

### 2.11 ROS

*In vitro* polarized monocytes were detached using PBS/1mM EDTA. The cells were washed with PBS and resuspended in 100µl RPMI-1640 supplemented with 5% NHS (Sigma-Aldrich). The cells were transferred to a black 96-well plate (Thermo Fisher) and placed in a 37°C pre-heated plate reader (VICTOR^3^, 1420 Multilabel Counter, PerkinElmer Life Sciences) for 10min. Next, 10µM H_2_DCFDA was added to each well and the plate was analysed at different time points up to 1hr of incubation. The plate was read at 485/535nm and analysed using the Wallac 1420 software (version 3.0, PerkinElmer Life Sciences) and Microsoft Excel. Unstained serum- and synovial fluid polarized monocytes served as controls.

### 2.12 Phosphorylation profiler array

From one healthy donor, 7x10^6^ isolated monocytes were exposed to 20% SF from n=4 patients with oligoarticular JIA or 20% of the monocyte donor’s serum (as a control) for 10min at 37°C. Next, cells were washed once with ice-cold PBS. Cells were then lysed, and a membrane-based 37 kinase phosphorylation array (R&D Systems) was performed according to the manufacturer’s instructions. The array was analysed by a ChemiDoc XRS+ (BioRad) and the ImageLab Software 5.1 Beta. The background was subtracted from the intensity of each dot, duplicates were averaged and the fold change of the SF- *vs.* serum- samples was calculated.

### 2.13 Liquid-chromatography mass spectrometry

Healthy monocytes from three donors were isolated using the pan monocyte isolation kit (Miltenyi) according to the manufacturer’s instructions and polarized overnight as described above with a serum pool (control) or SF pool from 6 oJIA patients. All conditions were performed in triplicates. Monocytes were detached using ice-cold PBS/1mM EDTA and gentle pipetting and washed two times with PBS. They were next lysed using cold RIPA buffer (Thermo Scientific) supplemented with cOmplete protease inhibitor cocktail (Roche) for 30min, 4°C on rotation. Next, the samples were treated with 5µM dithiothreitol (DTT) and incubated at 56°C, 30min followed by alkylation using 10µM iodoacetamide (IAA) for 30min, RT in the dark. Proteins were precipitated in 90% EtOH over night at -20°C. The following day, the samples were centrifuged at 14000g, 4°C for 10min. The supernatant was discarded, and the pellets were dried using a SpeedVac. The samples were resuspended in 100µl of 0.1M ammonium carbonate buffer and the protein concentrations were determined at 280nm using a NanoDrop (DS-11 Series Spectrphotometer/Fluormeter, DeNovix) and trypsination (Sequencing grade modified trypsin, porcine, Promega) was performed (1:50 trypsin:protein ratio) overnight at 37°C. Next day, trypsin was inhibited with 0.4% trifluoroacetic acid (TFA) and the peptides were dried by SpeedVac and stored at -80°C until use.

The samples were resolved in 22µl 2% ACN and 0.1% TFA and peptide concentration were determined at 215nm using NanoDrop. The samples were diluted to 0.5µg/µl and 2µl was injected to Liquid chromatography mass spectrometry (LC-MS). The LC-MS detection was performed on Tribrid mass spectrometer Fusion equipped with a Nanospray Flex ion source and coupled with an EASY-nLC 1000 ultrahigh pressure liquid chromatography (UHPLC) pump (Thermo Fischer Scientific). Peptides were concentrated on an Acclaim PepMap 100 C18 precolumn (75μm x 2cm, Thermo Scientific, Waltham, MA) and then separated on an Acclaim PepMap RSLC column (75μm x 25cm, nanoViper, C18, 2μm, 100Å) at the temperature of 45°C and with a flow rate of 300nl/min. Solvent A (0.1% formic acid in water) and solvent B (0.1% formic acid in acetonitrile) were used to create a nonlinear gradient to elute the peptides. For the gradient, the percentage of solvent B was maintained at 3% for 3min, increased from 3% to 30% for 90min and then increased to 60% for 15min and then increased to 90% for 5min and then kept at 90% for another 7min to wash the column.

The Orbitrap Fusion was operated in the positive data-dependent acquisition (DDA) mode. The peptides were introduced into the LC-MS *via* stainless steel Nano-bore emitter (OD 150µm, ID 30µm) with the spray voltage of 2 kV and the capillary temperature was set to 275°C. Full MS survey scans from m/z 350-1350 with a resolution of 120,000 were performed in the Orbitrap detector. The automatic gain control (AGC) target was set to 4 × 10^5^ with an injection time of 50ms. The most intense ions (up to 20) with charge states 2-5 from the full scan MS were selected for fragmentation in the Orbitrap. The MS2 precursors were isolated with a quadrupole mass filter set to a width of 1.2m/z. Precursors were fragmented by high-energy collision dissociation (HCD) at a normalized collision energy (NCE) of 30%. The resolution was fixed at 30000 and for the MS/MS scans, the values for the AGC target and injection time were 5 × 10^4^ and 54ms, respectively. The duration of dynamic exclusion was set to 45s and the mass tolerance window was 10ppm.

The raw DDA data were analysed with Proteome Discoverer™ 2.5 Software (Thermo Fisher Scientific). Peptides were identified using SEQUEST HT against UniProtKB human database (SwissProt TaxID=9606_and_subtaxonomies). The search was performed with the following parameters applied: static modification: cysteine carbamidomethylation and dynamic modifications: N-terminal acetylation and methionine oxidation. Precursor tolerance was set to 15 ppm and fragment tolerance was set to 0.05ppm. Up to 2 missed cleavages were allowed and Percolator was used for peptide validation at a q-value of maximum 0.01. Extracted peptides were used to identify and quantify them by label-free relative quantification. The extracted chromatographic intensities were used to compare peptide abundance across samples. Protein abundances were normalized against total amount of peptides.

The data was subsequently analysed in Microsoft Excel. The raw data is available *via* ProteomeXchange with identifier PXD033983. First, proteins identified with 0-2 unique peptides were excluded from further analysis. Next, data was filtered to include proteins found in ≥2 donors, and in ≥2 of the triplicates. The proteins fulfilling both criteria were used for statistical analysis, where the abundance values of the triplicates were averaged followed by paired t-test. In parallel, the values of the three donors were averaged, resulting in 2 groups, one for serum polarized monocytes and one for SF-polarized monocytes. These values were used to calculate the Log2 fold change. Proteins were considered upregulated if they had a log2 fold change of ≥1 and a p-value of <0.05, and downregulated if they had a log2 fold change of ≤ -1 and a p-value of <0.05.

Proteins that fulfilled the criteria (proteins found in ≥2 donors, and in ≥2 of the triplicates) for serum, but did not fulfil them for SF, were considered downregulated and combined with the proteins above. On the contrary, proteins that fulfilled the criteria for synovial fluid, but not serum, was considered upregulated and combined with the upregulated proteins. These proteins were then used for enrichment analysis (http://geneontology.org/) of altered biological processes using the PANTHER overrepresentation test (release 2022-02-02) and the GO Ontology database (DOI: 10.5281/zenodo.6399963, release 2022-03-22). Identified processes were considered if they had a false discovery rate adjusted p-value of <0.05 and ≥9 of the altered proteins included in the process. If several processes from the same gene ontology hierarchal tree were enriched, the most distal one with the lowest p-value was elected for analysis.

### 2.14 Transwell migration assay

Primary human fibroblast-like synoviocytes (FLS) from the knee (Cell Applications) were allowed to attach to the underside of 5µm pore-sized transwell inserts (Corning) in synoviocyte growth medium (Cell Applications) before the inserts were turned and placed in a 24-well plate (Corning) and cultured for 3-5 days. HMEC endothelial cells (ATCC) were subsequently added to the inside of the inserts and cultured in MCDB 131 medium (Gibco) supplemented with 10% foetal bovine serum, 10ng/ml hEGF, non-essential amino acids, sodium pyruvate and PenStrep for 72hrs before use.

The prepared inserts were placed in a new 24-well plate (Corning) with 400µl MCDB-131 medium supplemented with 20% synovial fluid (instead of 10% FBS). 0.2x10^6^ recently isolated monocytes from healthy donors (described above) were added to the inserts, and monocytes were allowed to migrate for 3hrs at 37°C, 5% CO_2_ followed by removal of the inserts. As a control, monocytes were instead added directly to wells containing the same medium and synovial fluids, but without inserts. The cells were incubated overnight at 37°C, 5% CO_2_. Next day, monocytes were detached, counted, and used for surface marker analysis (CD14, MerTK, CD86, HLA and CD16 (clone 3G8, APC-H7, BD) all diluted 1:200 and T-cell proliferation assays as described above.

### 2.15 Co-culture with fibroblast-like synoviocytes

Primary healthy FLS were seeded in 96-well plates (Falcon) and grown for 72hrs in synoviocyte growth medium before use. Monocytes were isolated from healthy donors and 1x10^5^ cells, in 100µl RPMI-1640 medium containing 20% SF, were added to the FLS after two washes with RPMI-1640. Wells with monocytes and synovial fluid but without FLS served as controls. The cells were incubated overnight at 37°C, 5% CO_2_. Next day, monocytes were detached, counted, and used for surface marker analysis (CD14, MerTK, CD86, HLA and CD16 all diluted 1:200, and T-cell proliferation assays as described above).

### 2.16 Statistics

Flow cytometry data was analysed using the CytExpert software (v2.3) or Kaluza (v2.1, both Beckman Coulter). Data is presented as median with interquartile range if not otherwise stated. Paired data was analysed using the Wilcoxon matched pairs signed rank test and unpaired data with the Mann-Whitney U test. In cases of multiple comparisons, correction was performed using Bonferroni correction unless otherwise stated. The ratio of T-cell proliferation was calculated using one sample Wilcoxon signed-rank test and the hypothetical median of 1. Some data was calculated using one-way ANOVA. Mass spectrometry data was analysed as described above. R programming language (27) was used to cluster the patients based on their joint markers into two groups by hierarchical clustering with “ward.D2” linkage function. A random forest predictive model based on the blood markers estimated the importance of variables by evaluating mean decrease accuracy when dropping every variable from the model. p<0.05 was considered statistically significant. All other data was analysed using GraphPad Prism 9 and Microsoft Excel.

## 3 Results

### 3.1 Synovial monocytes from oligoarticular JIA patients display pathogenic alterations, such as stimulation of T-cell activation, increased efferocytosis, and show signs of previous activation

**Figure 1:**
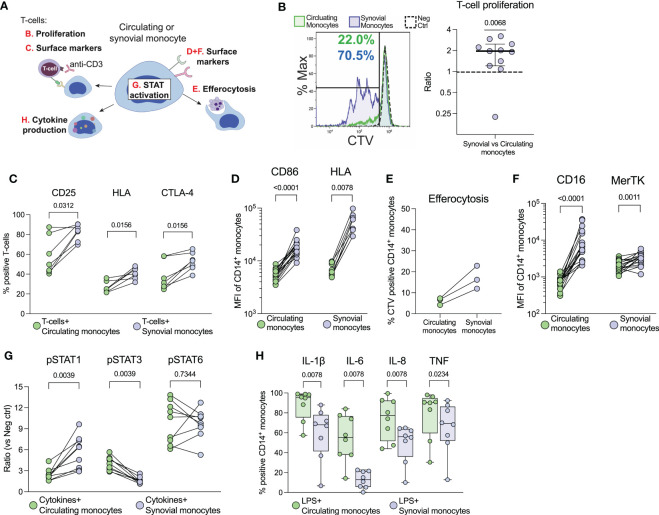
Synovial monocytes from patients with oJIA induce T-cell proliferation, display increased efferocytosis and a resistance to further activation. **(A)** Schematic overview of the various analyses of synovial- *vs.* circulating monocytes. **(B)** T-cells were isolated from healthy controls and stained with CellTrace Violet (CTV). They were subsequently activated with anti-CD3 and co-cultured with isolated monocytes for 72hrs, before being analysed for proliferation (presented as the ratio of percent proliferation induced by synovial- *vs.* circulating monocytes, line at median with IQR, n=11) and **(C)** expression of activation markers on T-cells by flow cytometry (n=7). **(D)** Shows expression of markers related to antigen presentation on monocytes [CD86 (n=17) and HLA (n=8)]. **(E)** As a measurement of clearance, isolated monocytes were incubated with CTV-stained apoptotic neutrophils (n=3), and the percent of CTV-positive CD14^+^CD66b^-^ monocytes is presented (reflecting uptake of neutrophils). **(F)** Shows expression of markers related to clearance on monocytes (CD16 and MerTK, n=17). **(G)** Monocytes in synovial fluid or blood were activated with IFNγ, IL-4 and IL-6 and investigated for their phosphorylation response of STATs (n=9). Values represent the ratio between stimulated *vs.* unstimulated samples. **(H)** Cytokine production of monocytes was studied intracellularly by incubation with golgiplug, followed by LPS activation (n=8). Statistics were performed using one sample Wilcoxon signed-rank test (with the hypothetical median of 1) or Wilcoxon matched pairs signed rank test. *oJIA, Oligoarticular juvenile idiopathic arthritis; MFI, Median fluorescence intensity; IFN, Interferon; IL, Interleukin; STAT, Signal transducer and activator of transcription; CTV, Cell Trace Violet; LPS, Lipopolysaccharide; TNF, Tumour necrosis factor*.

To investigate if monocytes from the joints of oJIA patients are functionally affected, we obtained synovial fluid (SF) and blood, to compare synovial monocytes to paired circulating monocytes (**Figure 1A**). When co-cultured with healthy T-cells, synovial monocytes displayed an increased ability to induce proliferation (p=0.0068) and activation markers in T-cells (CD25 (p=0.0312), HLA and CTLA-4 (both p=0.0156)) compared to circulating monocytes (**Figures 1B, C**). In parallel, synovial monocytes had increased expression of markers related to antigen presentation (CD86 (p<0.0001) and HLA, (p=0.0078, **Figure 1D**). Thus, synovial monocytes may contribute to prolongating synovial inflammation through linking innate immunity with adaptive immunity.

Patient synovial monocytes also displayed increased efferocytosis of apoptotic neutrophils (n=3, **Figure 1E**), as well as increased expression of surface markers related to clearance (CD16, p<0.0001 and MerTK, p=0.0011 **Figure 1F**). Hence, synovial monocytes also display regulatory mechanisms of increased clearance of apoptotic material. Finally, we investigated how patient synovial monocytes responded to activation using cytokines (IFNγ, IL-6 and IL-4) or LPS. The cells were primed for STAT1- (p=0.0039), resistant to STAT3- (p=0.0039) and had unchanged STAT6- (p=0.7344) phosphorylation compared to circulating monocytes (**Figure 1G**) and displayed resistance to cytokine production of IL-1ß, IL-6, IL-8 (all p=0.0078) and TNF (p=0.0234) following activation with LPS (**Figure 1H** **)**. Thus, synovial monocytes show signs of exhaustion and previous activation. Taken together, these data highlight a functional imbalance of monocytes in the pathogenesis; as they (1): drive inflammation through T-cell activation (2), are primed for STAT1- and resistant to STAT3 activation (3), display increased clearance of apoptotic cells and (4) show resistance to cytokine production.

### 3.2 Synovial fluid polarization induces a regulatory, pro-clearance phenotype and downregulates co-stimulatory capabilities in healthy monocytes

**Figure 2:**
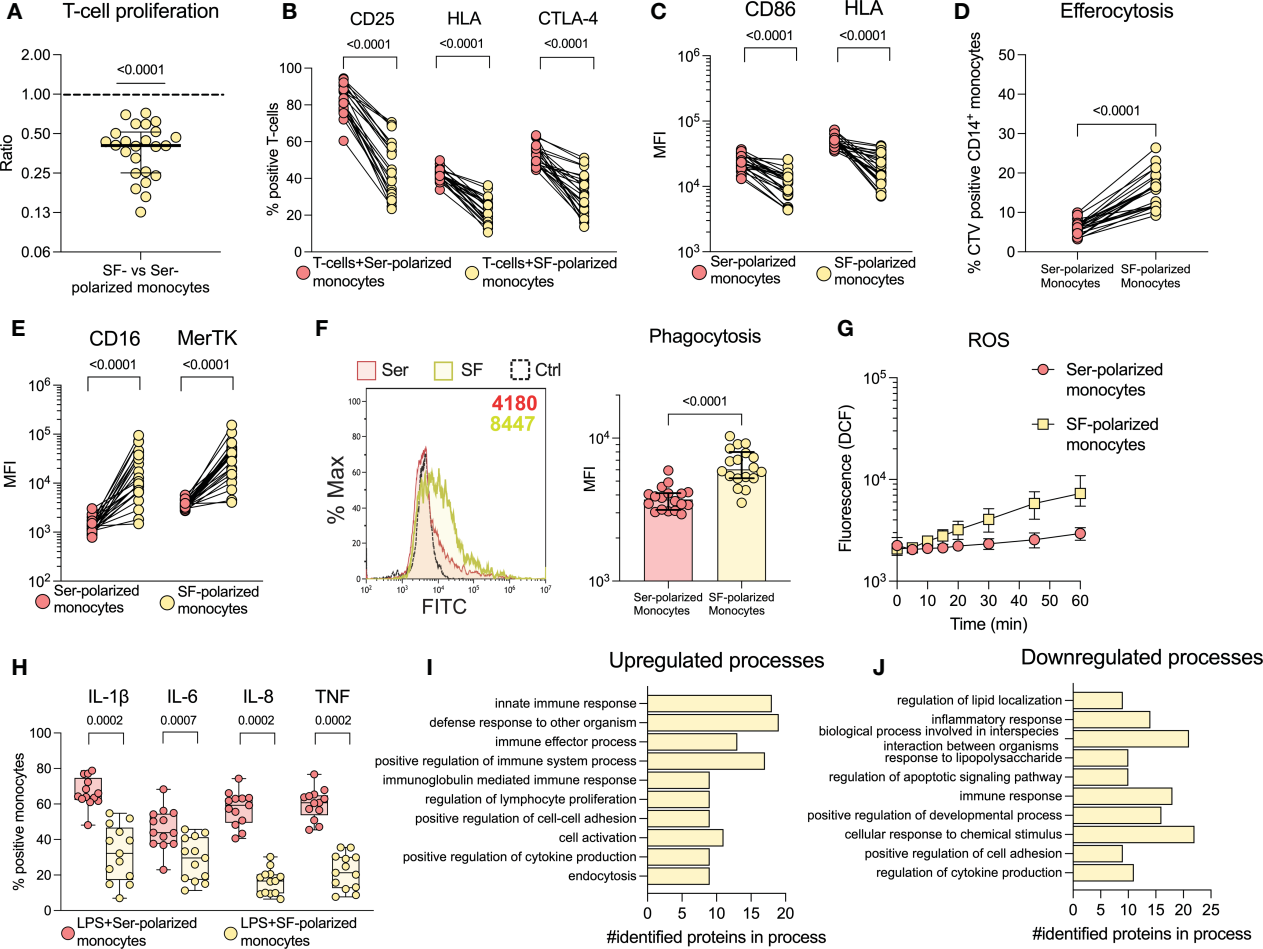
Synovial fluid induces a regulatory phenotype in healthy monocytes and downregulates co-stimulatory capabilities. Monocytes were isolated from healthy controls and polarized with 20% serum (Ser) or 20% synovial fluid (SF) overnight. Polarized monocytes were co-cultured with CellTrace violet (CTV) stained healthy CD3-activated T-cells and analysed by flow cytometry for **(A)** proliferation (n=24, data is presented as the ratio of proliferation induced by SF-polarized monocytes *vs.* serum-polarized monocytes) and **(B)** activation markers on T-cells (n=24). **(C)** Shows expression of CD86 and HLA in monocytes (n=24). **(D)** Monocytes were incubated with CTV-stained apoptotic neutrophils and analysed for uptake of these cells. The percentage of CTV positive CD14^+^CD66b^-^ monocytes is shown (n=21). **(E)** Displays the expression of CD16 and MerTK in monocytes. **(F)** Phagocytosis of polarized monocytes was assessed following incubation with opsonized FITC labelled beads (n=18). **(G)** ROS production of polarized monocytes stained with H_2_DCFDA at different time points (n=24, median with interquartile range). **(H)** Cytokine production of polarized monocytes was studied intracellularly following LPS activation (n=13). Changes in monocyte proteomics were analysed (n=3) through biological process enrichment, and the top 10 **(I)** upregulated and **(J)** downregulated processes following polarization are presented. Statistical analyses were performed using one sample Wilcoxon signed-rank test (with the hypothetical median of 1) or Wilcoxon matched pairs signed rank test. Lines at median with IQR*. MFI, Median fluorescence intensity; IL, Interleukin; CTV, Cell Trace Violet; LPS, Lipopolysaccharide; ROS, Reactive oxygen species; TNF, Tumour necrosis factor*.

To investigate the effect of SF on the function and phenotype of monocytes, we cultured healthy monocytes overnight with SF or paired serum (as a control). Monocytes polarized with SF induced less proliferation (p<0.0001) and activation markers (p<00001) in healthy T-cells compared to serum polarized monocytes (**Figures 2A, B**). Accordingly, SF-polarized monocytes expressed less CD86 and HLA (p<0.0001, **Figure 2C**). In addition, SF-polarization resulted in an increased uptake of apoptotic neutrophils (p<0.0001, **Figure 2D**) as well as elevated expression of markers related to clearance (CD16 and MerTK, p<0.0001, **Figure 2E**). Serum from oJIA patients did not induce a similar phenotype as SF when compared to normal human serum (NHS, **Supplementary Figure 5** **)**. Instead, compared to NHS, oJIA serum induced a minor but significant downregulation of CD16 (p=0.0368), as well as upregulation of CD86 (p=0.0102) and HLA (p=0.0104). We have previously shown that synovial monocytes have reduced capacity to phagocytose and produce ROS (19). Here, we instead observed that SF-polarization induced an increase in phagocytosis (p<0.0001, **Figure 2F**) and ROS production over time in healthy monocytes (**Figure 2G**). Finally, monocytes polarized with SF produce less pro-inflammatory cytokines (IL-1ß, IL-8, TNF (p=0.0002) and IL-6 (p=0.0007) upon LPS activation **Figure 2H**). These results suggest that SF from oJIA patients downregulates antigen presentation capabilities and induce a regulatory phenotype in healthy monocytes, mimicking some of the features of the patients’ synovial monocytes.

### 3.3 Biological processes involved in immune- and regulatory processes are upregulated by synovial fluid

To explore the effect of SF at a broader scale, we analysed healthy monocytes stimulated with SF by liquid-chromatography mass spectrometry. We identified 62 upregulated- and 66 downregulated proteins (see **Supplementary Figure 6** for details on selection). All the differentially regulated proteins can be found in **Supplementary Table 1**. To understand the involvement of these proteins, we performed enrichment analysis of biological processes using gene ontology. The top 10 enriched biological processes of regulated proteins are presented in **Figures 2I, J**. Generally, the upregulated processes are immune- and regulatory processes, such as innate immune response, and regulation of immune effector process, lymphocyte proliferation and cell-cell adhesion. The downregulated processes are involved in lipid localization, inflammation, and regulation of apoptosis. These findings support our previous results and suggest that SF induces upregulation of immune- and regulatory processes.

### 3.4 Synovial fluid predominately induces STAT3 phosphorylation through IL-6 signalling

**Figure 3:**
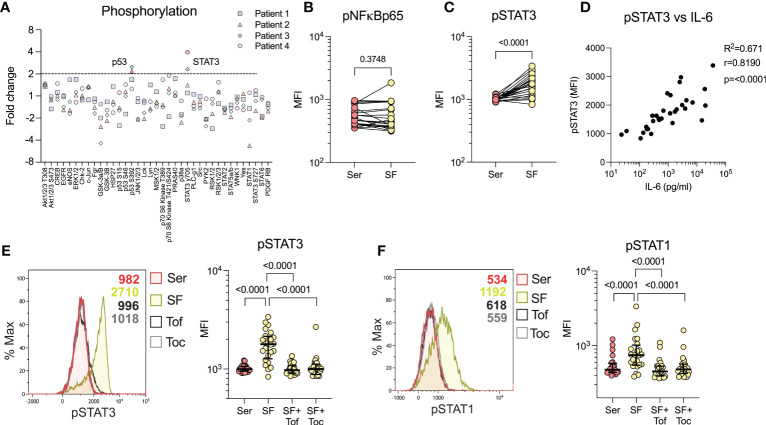
Synovial fluid predominately induces STAT3 phosphorylation through IL-6. **(A)** Broad spectrum phosphorylation array of 37 kinases following 20% synovial fluid (SF) stimulation of healthy monocytes compared to serum (n=4). **(B)** Analysis of NFkBp65 phosphorylation with SF *vs.* serum (Ser) stimulation by flow cytometry (n=24). **(C)** Analysis of STAT3 phosphorylation with SF (n=24) by flow cytometry. **(D)** Shows correlation between levels of STAT3 phosphorylation in monocytes versus IL-6 in the SF (n=33, Spearman correlation). **(E)** SF stimulation and STAT3 phosphorylation analysis, with pre-treatment of monocytes with tofacitinib (1µM) or tocilizumab (100ng/ml) and **(F)** STAT1 phosphorylation (n=24). Data is presented as median with IQR. *MFI, Median fluorescence intensity; IL, Interleukin; STAT, Signal transducer and activator of transcription; Tof, Tofacitinib; Toc, Tocilizumab*.

To characterize the major signalling pathways induced by SF, we performed a broad-spectrum phosphorylation assay, using healthy monocytes and SF from n=4 patients. SF predominately induced phosphorylation of STAT3 (pSTAT3) and, to a lesser degree, p53, compared to serum (**Figure 3A**). In addition to the proteins involved in the broad-spectrum phosphorylation assay, we were interested in NFκB, a downstream signalling transducer of TNF. NFκB phosphorylation was investigated separately by flow cytometry, where we could not detect an increased phosphorylation in cells stimulated with SF compared to paired serum (p=0.3748, **Figure 3B**). As STAT3 is involved in immune function, we continued to investigate this factor and could confirm the findings that SF induces pSTAT3 in a larger sample population (n=24, p<0.0001, **Figure 3C**). Next, we reanalysed the IL-6 levels in SF, as published previously (19), and observed a strong correlation between IL-6 and pSTAT3 (n=31, spearman r=0.819, p<0.0001, **Figure 3D**). To confirm that IL-6 is responsible for pSTAT3, we used two inhibitors of the JAK/STAT signalling pathway: the anti-IL-6-R antibody tocilizumab and the small molecule inhibitor of JAK, tofacitinib. Both tocilizumab and tofacitinib fully inhibited pSTAT3 (p<0.0001) and pSTAT1 (p<0.0001), both of which are induced by IL-6 (**Figures 3E, F**). Taken together, these data suggest that SF predominately induces monocyte activation *via* STAT1/3 phosphorylation in healthy monocytes through an IL-6 driven mechanism.

### 3.5 Blocking of IL-6/JAK/STAT signalling inhibits several aspects of the synovial fluid polarized phenotype

**Figure 4:**
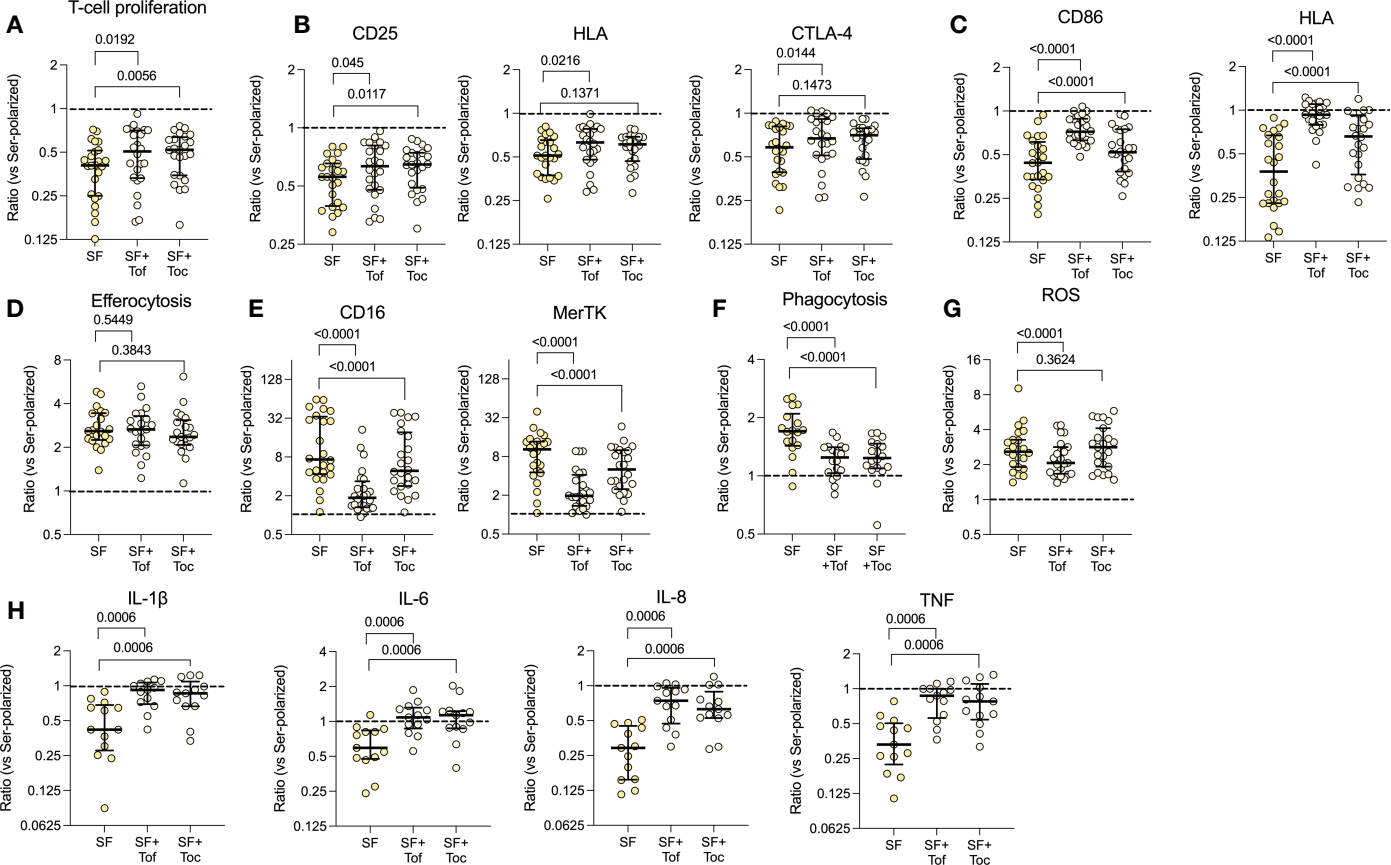
The IL-6/JAK/STAT axis is responsible for several of the phenotypical effects induced by synovial fluid. Healthy monocytes were pre-treated with or without tofacitinib (Tof, 1µM) or tocilizumab (Toc, 100ng/ml) and then polarized with 20% serum (Ser) or 20% synovial fluid (SF). Polarized monocytes were incubated with healthy CellTrace Violet (CTV) stained anti-CD3 activated T-cells for 72hrs and assessed for **(A)** proliferation of T-cells (n=24) and **(B)** activation markers on the T-cells. **(C)** Shows expression of CD86 and HLA in polarized monocytes (n=24). **(D)** Efferocytosis of CTV-stained apoptotic neutrophils by CD14^+^CD66b^-^ monocytes (n=21). **(E)** CD16 and MerTK expression in polarized monocytes. **(F)** Phagocytosis of opsonized FITC labelled beads (n=18). **(G)** Shows ROS production after 1hr of incubation following H_2_DCFDA staining (n=24). **(H)** Intracellular cytokine production following pre-incubation with golgiplug and LPS activation for 4 hrs (n=13). Data represents the ratio *vs.* serum-polarized monocytes, and the dotted lines represents a ratio of 1:1. Full lines at median with IQR, Wilcoxon matched pairs signed rank test. *ROS, reactive oxygen species; CTV, Cell Trace Violet; LPS, Lipopolysaccharide; MFI, Median fluorescence intensity; IL, Interleukin; STAT, Signal transducer and activator of transcription; Tof, Tofacitinib; Toc, Tocilizumab; LPS, Lipopolysaccharide; TNF, Tumor necrosis factor*.

To investigate the role of IL-6/JAK/STAT on the functional alterations observed in the patients’ monocytes, we pre-treated healthy monocytes with tocilizumab or tofacitinib, followed by polarization with SF. Tocilizumab (p=0.0056) and tofacitinib (p=0.0192) restored the decreased T-cell proliferation to some extent, and had mild effects on the T-cell expressed activation markers (**Figures 4A, B**). Yet, the CD86 and HLA expression was restored upon inhibition of IL-6/JAK/STAT (p<0.0001, **Figure 4C**). Neither drug had any substantial effect on the increased efferocytosis (p=0.5449 and p=0.3843, **Figure 4D**), but efficiently restored the CD16 and MerTK expression, although tofacitinib seemed more efficient (all p<0.0001, **Figure 4E**). Both drugs also inhibited the increased phagocytosis (p<0.0001, **Figure 4F**), and tofacitinib partly inhibited the increased ROS production, but not tocilizumab (p<0.0001, **Figure 4G**). Finally, the SF induced resistance to LPS activated cytokine production was inhibited by both drugs (all p=0.0006, **Figure 4H**). These results suggest that the IL-6/JAK/STAT pathway is responsible for several of the regulatory features observed in synovial monocytes and highlight potential targets of anti-IL-6/JAK/STAT therapy.

### 3.6 Cell-cell interactions induce increased antigen presentation capabilities in synovial fluid polarized monocytes

**Figure 5:**
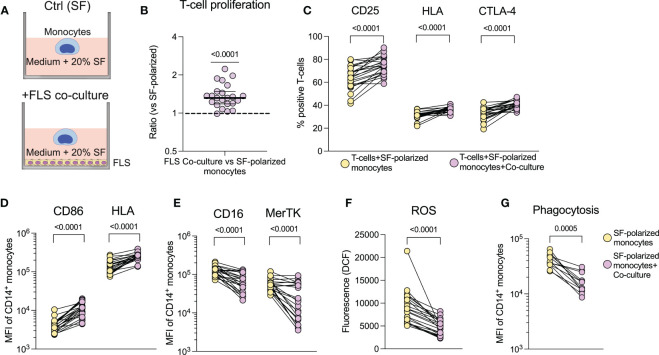
Increased co-stimulatory capabilities of synovial monocytes are induced *in vitro* in healthy monocytes through co-culture with fibroblast-like synoviocytes. **(A)** Experimental setup of the co-culture assay. **(B)** Monoculture of monocytes (SF) and monocytes co-cultured with FLS (Co-culture) were detached following overnight culture and seeded with CellTrace Violet (CTV) stained anti-CD3 activated T-cells, which were cultured (1:10 monocytes to T-cells) for 72hrs, followed by analysis of proliferation (displayed as ratio of percent proliferation between FLS co-culture *vs.* monoculture of monocytes) and **(C)** expression of activation markers in T-cells (n=23). **(D)** Shows changes in surface expression of CD86 and HLA and **(E)**, of CD16 and MerTK. **(F)** Displays ROS production after 1hr incubation following H_2_DCFDA staining (n=23) and **(G)** phagocytosis of opsonized FITC labelled beads (n=12). Statistics were performed using one sample Wilcoxon signed-rank test (with the hypothetical median of 1) or Wilcoxon matched pairs signed rank test. Lines at median with IQR*. FLS, Fibroblast,like synoviocytes*; *ROS, Reactive oxygen species; MFI, Median fluorescence intensity; SF, Synovial fluid*.

SF polarization of healthy monocytes does not replicate the increased co-stimulatory capabilities observed in the patients’ synovial monocytes; nor does it induce a decrease in ROS production and phagocytosis, as previously observed (19). We have recently shown that migration through a transwell system influences the effector functions of neutrophils (20). To determine how the patient’s synovial monocytes may acquire the remaining features, we investigated the effect of migration through an artificial synovial membrane towards SF on healthy monocytes (**Supplementary Figure 7A**). We show that migration induced some, but not all, of the features of the patients’ monocytes, such as increased CD86 expression and increased T-cell proliferation (**Supplementary Figures 7B–F**). We theorized that the monocytes may instead require prolonged cell-cell contact to induce the remaining features. Thus, we studied the effect of co-culture between monocytes and FLS in SF-polarizing environment (**Figure 5A** **)**. Co-culture resulted in an increased ability of the monocytes to induce T-cell activation (p<0.0001, **Figures 5B, C**). Additionally, these monocytes had increased expression of both CD86 and HLA (p<0.0001, **Figure 5D**) as well as a decreased expression of CD16 and MerTK (p<0.0001, **Figure 5E**). Furthermore, they also display reduced ROS production (p<0.0001, **Figure 5F**) and phagocytosis (p=0.0005, **Figure 5G**). Finally, tofacitinib and tocilizumab did not inhibit the increased expression of CD86, HLA or T-cell proliferation, but rather trended to increase it further, in both migrated and co-cultured monocytes (**Supplementary Figure 8**). Thus, co-culture with FLS induces the remaining features in healthy monocytes that are observed in the patients’ synovial monocytes, such as increased co-stimulatory capabilities. These data suggest that cell-cell contact promotes inflammatory monocytes and highlight the monocytes’ potential to drive adaptive immune responses.

### 3.7 The IL-6/JAK/STAT axis activation in monocytes is reflected in circulating markers of inflammation

**Figure 6:**
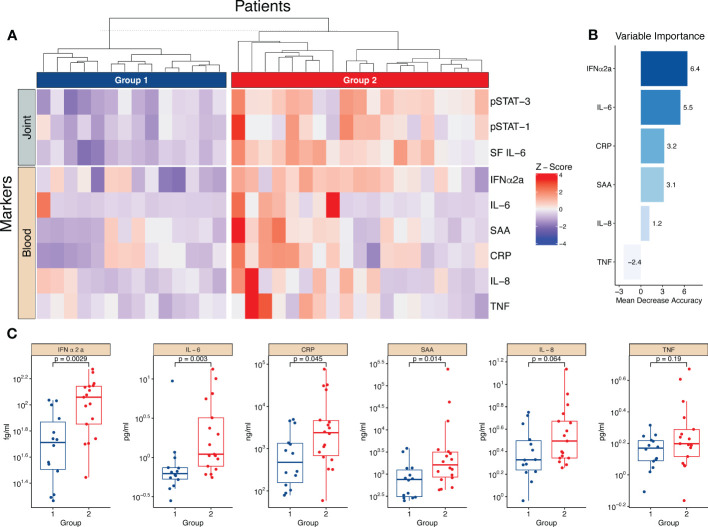
The magnitude of the synovial IL-6/JAK/STAT axis is reflected in several circulating markers of inflammation. **(A)** Heatmap showing hierarchical clustering into two groups based on three parameters, synovial (joint) IL-6 levels and monocyte pSTAT1- and pSTAT3 activation. It further shows the distribution of circulating (blood) markers of inflammation measured in plasma. **(B)** Shows the importance score of the circulating markers in predicting the two groups based on a random forest predictive model. **(C)** Displays the statistical difference between group one and two regarding the levels of circulating markers. Statistical analyses between groups were performed using Mann-Whitney U test. *SF, Synovial fluid*; *IFN, Interferon; CRP, C,reactive protein; SAA, Serum amyloid A; TNF, Tumor necrosis factor*.

Given the effect of IL-6/JAK/STAT on the monocyte phenotype, we speculated that the synovial IL-6/JAK/STAT driven inflammation is reflected in the blood. Using hierarchical clustering based on three parameters, synovial IL-6, pSTAT1- and pSTAT3, we generated two different groups of low- *vs.* high synovial IL-6 driven inflammation. Representative histograms of pSTAT1 and pSTAT3 expression of a patient in group 1 and a patient in group 2 can be found in **Supplementary Figure 9A**. We subsequently depicted Z-score pattern of several prominent markers of inflammation in plasma (IFNα2a, IL-6, SAA, CRP, IL-8, and TNF) to highlight any differences between the two groups (**Figure 6A**). For exploratory purposes, we next used a random forest model using the plasma markers to predict the groups. IFNα2a and IL-6 were ranked the highest in the variable importance scores (**Figure 6B**). Thereafter, we analysed the differential expression of the circulating markers between the two groups, and we observed that most markers were significantly elevated in the group with prominent synovial IL-6/JAK/STAT driven inflammation (**Figure 6C**). Clinically, there were no major differences between the two groups, although patients in group two tended to be younger and had an overrepresentation of males (**Supplementary Figures 9B, C**). These results suggest that the magnitude of synovial inflammation related to the IL-6/JAK/STAT axis is reflected in the circulation, and the possibility to identify patients more relevant for anti-IL-6/JAK/STAT therapy. Notably, this is apparent in several different circulating markers.

## 4 Discussion

**Figure 7:**
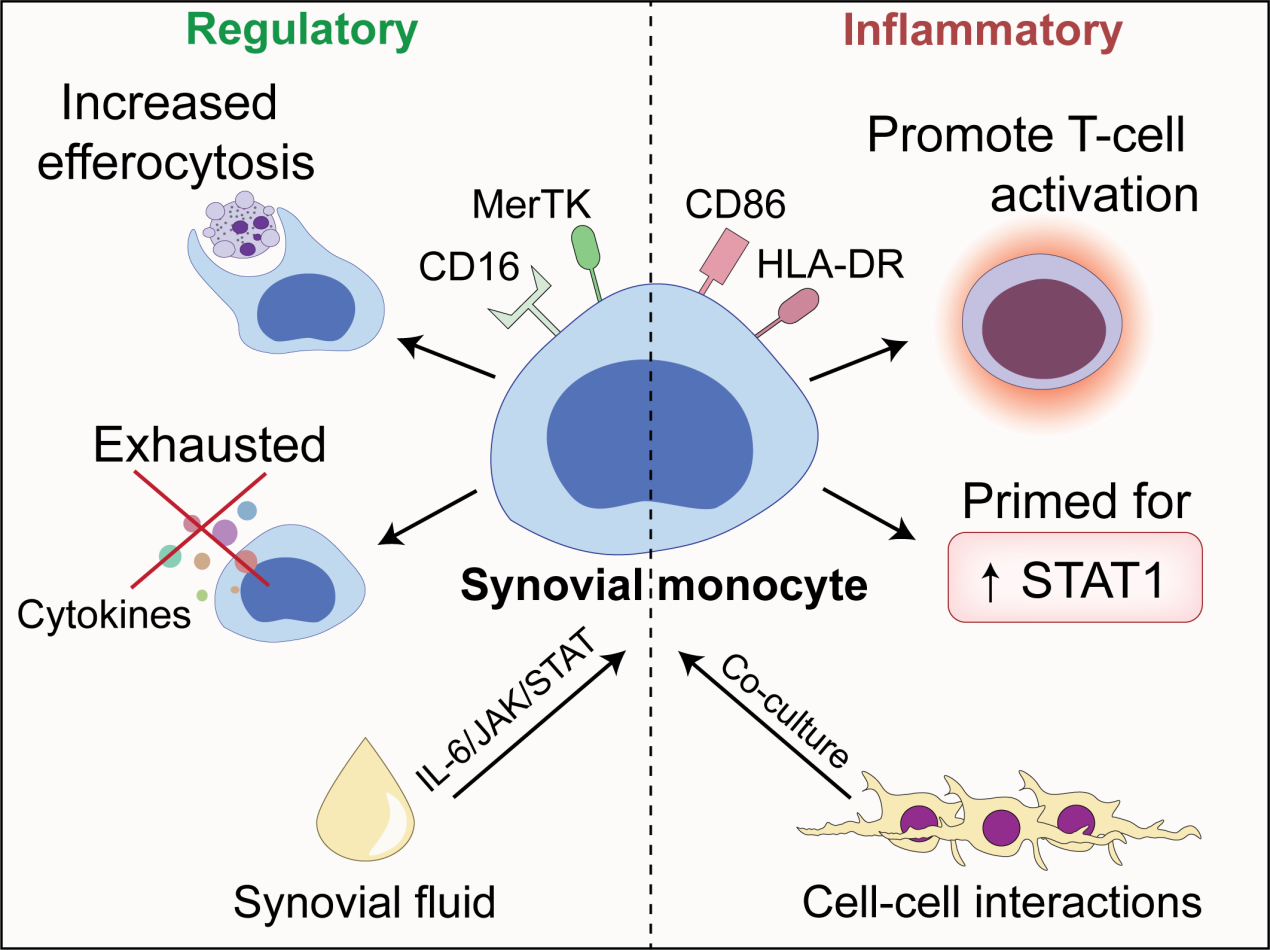
An illustrative overview of the main findings presented in this paper.

Monocytes are important cells of the innate immune system with the potential to drive inflammation. Here, we show that synovial monocytes from patients with untreated childhood-onset arthritis (oligoarticular juvenile idiopathic arthritis, oJIA) have altered functions with implications in both inflammatory- and regulatory processes (**Figure 7**). The regulatory aspects can be replicated *in vitro* through polarization of healthy monocytes with synovial fluid (SF), which is mainly driven through an IL-6/JAK/STAT mechanism. The magnitude of IL-6/JAK/STAT activation is also reflected in circulating markers of inflammation. The co-stimulatory aspects are induced by cell-cell interactions (i.e., co-culture with fibroblast-like synoviocytes (FLS)). These data provide insight into the pathogenesis of arthritis and suggest that monocytes drive chronic inflammation, e.g., through prolongating T-cell responses. Interestingly, the magnitude of IL-6/JAK/STAT activation is reflected in circulating markers of inflammation, implying that we could identify a group of patients likely to benefit from IL-6/JAK/STAT inhibition, suggesting use in personalized medicine for the treatment of these individuals.

In adult-onset arthritis, monocytes are found in the inflamed synovium, expressing markers of activation, producing cytokines, and can activate other immune cells, such as T-cells (1, 7, 28). Less is known of monocytes in childhood-onset arthritis. We have previously shown that synovial monocytes in oJIA are polarized, displaying markers of activation, such as co-stimulatory molecules (19). A recent study found epigenetic alterations related to an activated phenotype in synovial monocytes from JIA patients, and proposed a role of blocking JAK/STAT to inhibit phenotype (22). Here, we showed that synovial monocytes are primed for STAT1-, and resistant to STAT3 phosphorylation following cytokine stimulation, suggesting a predisposition for further pro-inflammatory activation. The resistance to STAT3 phosphorylation could be due to exhaustion following prior exposure to IL-6, or due to downregulation of the IL-6 receptor/co-receptor (29).

It has recently been shown that synovial monocytes have an increased ability to induce T-cell proliferation (30). In addition, synovial T-cells display an activated phenotype and produce several pro-inflammatory cytokines, such as IL-17 (31–33). The role of monocytes is highlighted in several animal models (34, 35). Monocytes initially display an inflammatory phenotype with high expression of co-stimulatory markers but acquire regulatory features over time (34). This is similar to our results, were monocytes show a dual phenotype. Here, we confirmed that synovial monocytes express high levels of co-stimulatory markers. We further showed that the monocytes induce proliferation and activation markers in healthy T-cells. Notably, all markers investigated (CD25, HLA-DR and CTLA-4) were upregulated, suggesting general activation. Interestingly, several T-cell populations exist within the inflamed joint of oJIA patients, mainly being of a Th1-phenotype, which correlate with disease severity (36). Notably a Th1-like Treg population expressing CTLA-4, a negative regulator, was also identified (36). Furthermore, a recent study found a general PD-1 expression on clonally expanded synovial T-cells (37). These data support a complex role of T-cell populations in chronic inflammation, with both inflammatory and suppressive capabilities, and suggest that even though T-cells express checkpoint inhibitors, these receptors may not be activated. Moreover, we have recently shown that synovial neutrophils lose their capacity to suppress T-cell proliferation, and that healthy neutrophils lose this ability upon migration (20). Thus, an increase in activation of T-cells by monocytes, and a loss of suppressive capability by neutrophils may explain the expansion and activation of T-cells within the joint of these patients.

Notably, this is not an effect of the environment on monocytes alone, as SF polarization reduced the ability of monocytes to activate T-cells. Indeed, this reduced ability to induce T-cell activation is supported by our proteomics data, where regulation of lymphocyte proliferation was one of the top upregulated processes. This process included proteins such as VSIG4 and GPNMB, which are described to negatively regulate T-cell proliferation (38, 39). However, co-culture with healthy FLS, or migration to a certain extent, resulted in an increased ability of the monocytes to induce T-cell activation compared to SF polarization alone, suggesting a role of cell-cell interactions in driving the synovial monocyte phenotype. A link between monocytes and FLS is well established; monocytes can induce cytokine production in synoviocytes, such as IL-6, whilst synoviocytes produce factors such as GM-CSF, that in turn can induce HLA and increase viability in monocytes (40–42). A direct cell-cell contact may be important in the interaction of these cells and can induce factors such as MIP-1α (43). Finally, synoviocytes in JIA upregulate VCAM-1, which may facilitate leukocyte recruitment, attachment, and activation (29, 44). Speculating, cell-cell contact dependent activation mediated by molecules such as VCAM-1, or soluble factors such as GM-CSF, could be crucial in mediating monocyte activation. We are going to pursue the interaction between JIA FLS and monocytes in oJIA in future studies.

Synovial monocytes also displayed signs of a regulatory phenotype; features which could all be replicated *in vitro* following SF polarization, driven partly by IL-6/JAK/STAT3 signalling. This is in contrast to the patients’ synovial monocytes, which showed resistance to STAT3 phosphorylation. However, this is likely due to exhaustion, e.g., from prior exposure, or downregulation of the IL-6 receptor (29). IL-6/JAK/STAT signalling appears to have a central role in arthritis, where it is believed to drive recruitment, inflammation, and phenotypical changes in T-cells, B-cells and FLS (45–47). Indeed, STAT3 knockout is protective in a mouse model of arthritis; and tofacitinib treatment reduced synovial matrix metalloproteinases, chemokines, and phosphorylation of STAT3, which in turn correlated with clinical responses in rheumatoid arthritis (48, 49). A role of IL-6 in oJIA has been implied decades ago (50). In addition, a recent study investigating chromatin data in monocytes in JIA patients found that IL-6 binding was one of the top enriched processes (21). Interestingly, our data showed that the magnitude of IL-6/JAK/STAT activation in monocytes was reflected in the levels of several circulating cytokines. By clustering the patients into two groups based on the synovial IL-6/JAK/STAT axis, we observed that patients with high IL-6/JAK/STAT involvement also had significantly higher levels of several circulating cytokines, with IFNα2a and IL-6 being the most important variables in predicting the two groups. Notably, the impact of TNF was minor. Previous studies have also investigated the relationship between circulating- and synovial inflammatory markers (51, 52). These studies also suggest potential differences, both between and within JIA subgroups. Even though oJIA is not traditionally associated with systemic inflammation, our results further support that, following future studies, synovial or circulating markers could be important in treatment prediction, and the division of patients with a more IL-6 prominent disease.

Yet, in line with our results, the effect of IL-6/JAK/STAT signalling on monocytes/macrophages is believed to be primarily regulatory/anti-inflammatory (53–55). Here, blocking of IL-6/JAK/STAT with tocilizumab or tofacitinib restored several aspects of the phenotype induced by SF. There were some differences in the effect of these drugs, were tofacitinib generally displayed a stronger inhibition. This could potentially be attributed to tofacitinib being more efficient as it is a small molecule inhibitor, compared to tocilizumab, which is an antibody. Another reason could be that tofacitinib targets mainly JAK1/3, compared to IL-6 alone, thus blocking other cytokine signals as well. An impaired efferocytosis, resulting in accumulation of cellular debris and increased autoantigenic burden, can further autoimmune reactions (56). However, we did instead observe an increased efferocytosis of synovial monocytes and SF-polarized healthy monocytes. Moreover, SF polarization induced high MerTK expression compared to the patients’ synovial monocytes. This discrepancy could be due to cell interactions *in vivo*, e.g., with FLS, as co-culture resulted in reduced MerTK expression compared to SF alone. Additionally, our results showed that SF polarization also induced an increase in phagocytosis, whilst synovial monocytes in oJIA, as well as in RA, have a decreased ability to phagocytose (19, 57, 58). Though, this reduced phagocytosis in our patients could be replicated following co-culture with FLS. Thus, synovial monocytes might have impaired clearance of certain pathways, such as immune complexes, whilst remaining functional in others. Furthermore, we observed a reduction of pro-inflammatory cytokine production following activation with LPS, both in patients’ synovial monocytes and in *in vitro* SF-polarized monocytes, suggesting resistance to a pro-inflammatory response. This was dependent on IL-6 signalling *in vitro*, which has previously been described to impair LPS induced cytokine production (59). Given the presence of endogenous toll-like receptor (TLR) ligands in the joint and the differential expression of TLRs on synovial monocytes, IL-6 could play a role in regulating TLR induced cytokine production in the joint (1, 12, 59).

Importantly, regulatory monocytes likely still contribute to chronic inflammation. For example, regulatory monocytes and macrophages are prominent in angiogenesis, a process believed to be unfavourable in arthritis (9, 10). CD163, a classical marker of regulatory macrophages, is highly expressed in spondylarthritis compared to rheumatoid arthritis and associated with inflammation (5, 11, 60). Interestingly, the synovial tissue of these patients has more prominent vascularization (60). A similar observation has been made in JIA, were polyarticular patients present with lower vascularization compared to oligoarticular- or enthesitis-related ones (61). In addition, regulatory monocytes and macrophages are potent inflammatory cytokine producers in arthritis upon TLR- and immune complex stimulation (12). Taken together, our data suggests that SF mainly induces a regulatory phenotype in monocytes, but this phenotype may not be advantageous in chronic disease, and we suggest that it would be beneficial to restore synovial homeostasis blocking IL-6/JAK/STAT, rather than favouring anti-inflammation. In addition, treatment of the patients’ monocytes with JAK/STAT inhibitors reverses epigenetic changes, further supporting blockage of this pathway to restore homeostasis (22). Finally, blocking of monocytes from entering the joint, and subsequent cell-cell interactions, might represent another crucial mechanism.

It is likely that monocytes have a similar role in other arthritic diseases, although we did not include other diagnoses in this study. For example, synovial monocytes express similar surface markers and also drive T-cell activation in RA (7). However, there are also evidence of differences between arthritic diseases. For example, SF from RA patients induces a distinct phenotype in monocytes compared to spondyloarthritis patients (6). Additionally, CD163 positive monocytes have been described in the joint of enthesitis-related JIA, but not oJIA (19, 62). Taken together, these studies suggests both similarities and differences in the function of monocytes in arthritis.

There are some limitations to this study. There are several aspects not investigated in this study that may influence monocyte function *in vivo*, such as time spent in the joint, hypoxia, and biomechanical factors (e.g., tissue stiffness). In addition, macrophage replacement represents another important aspect of monocyte function. Monocyte-derived macrophages are believed to have a crucial role in adult arthritis and correlate with disease activity, thus representing an interesting area for future studies in oJIA (63, 64). Another limitation is the use of the membrane-based phosphorylation assay, which was utilized as an exploratory way of identifying potential pathways induced by SF. Even though STAT1 was included, it was not induced above the cut-off level, suggesting the potential overseeing of weaker inductions. Furthermore, even though the phosphorylation assay contained 37 common kinases, there are still numerous pathways not included, that could also contribute to the resulting phenotype. Indeed, blocking IL-6/JAK/STAT did not normalize all features induced by SF, especially when using with tocilizumab, suggesting a role of other pathways/factors in SF. Still, by identifying STAT3, we believe we were able to identify a major contributor the monocyte phenotype. The statistical changes in the proteomics analysis should be interpreted carefully, as we analysed 3 donors, the statistical power is low and false discovery rate could not be accounted for. Thus, it should be considered supportive rather than conclusive. Furthermore, we descriptively categorized patients into high and low synovial IL-6/STAT driven inflammation and compared the circulating cytokine levels between the two groups. Given the small sample size, we did not adjust for confounders and the results should be interpreted carefully. Finally, the T-cell interaction assays used in this study involves pan TCR activation using an anti-CD3 antibody. Thus, the extent to how monocytes contribute to inflammation by antigen presentation through MHC, and which antigens that are of importance, remains to be determined.

In conclusion, we show that synovial monocytes contribute to the pathogenesis in childhood-onset arthritis by driving chronic inflammation. Their phenotype could be replicated *in vitro* using SF, which induced regulatory aspects through IL-6/JAK/STAT signalling, and co-culture with FLS, which induced inflammatory properties. In addition, the synovial IL-6/JAK/STAT axis was reflected in circulating markers, suggesting a group of patients more likely to benefit from targeting the IL-6/JAK/STAT axis to restore synovial homeostasis.

## Data availability statement

The raw data supporting the conclusions of this article will be made available by the authors, without undue reservation. The datasets presented in this study can be found in online repositories. The names of the repository/repositories and accession number(s) can be found below: PXD033983 (ProteomeXchange).

## Ethics statement

The studies involving human participants were reviewed and approved by the Regional Ethical Review Board for southern Sweden (2016/128). Written informed consent to participate in this study was provided by the participants’ legal guardian/next of kin.

## Author contributions

TS conceptualized the study, designed experiments, analysed, and interpreted data, and wrote the manuscript. AM, SA-B, and ER performed experiments, interpreted data, and reviewed and revised the manuscript. EB, PK, AD, and BM collected clinical data and samples, interpreted data, and reviewed and revised the manuscript. SN analysed and interpreted statistical data and reviewed and revised the manuscript. AB and FK interpreted data and reviewed and revised the manuscript. RK conceptualized the study, collected clinical data and samples, interpreted data, and wrote the manuscript. All authors contributed to the article and approved the submitted version.
