# Supplementary material for: Synovial monocytes contribute to chronic inflammation in childhood-onset arthritis via IL-6/STAT signalling and cell-cell interactions
Source: Front Immunol. 2023 May 22;14:1190018. doi: 10.3389/fimmu.2023.1190018 (PMC10239926; doi:10.3389/fimmu.2023.1190018)
Supplement: Supplementary file 1 [file DataSheet_1.pdf]

Supplementary Material

Synovial monocytes contribute to chronic inflammation in childhood-onset arthritis via IL-6/STAT signaling and cell-cell interactions

Tobias Schmidt[1,3*], Alma Dahlberg[1,3], Elisabet Berthold[2], Petra Król[1,3], Sabine Arve-Butler[2,3], Emilia Rydén[1,3], Seyed Morteza Najibi[2], Anki Mossberg[1,3], Anders A. Bengtsson[2], Fredrik Kahn[4], Bengt Månsson[1] and Robin Kahn[1,3]

[1]Department of Pediatrics, Clinical Sciences Lund, Lund University, Lund, Sweden
[2]Department of Rheumatology, Clinical Sciences Lund, Lund University, Lund, Sweden
[3]Wallenberg Center for Molecular Medicine, Lund University, Lund, Sweden
[4]Department of Clinical Sciences, Division of Infection Medicine, Lund University, Lund, Sweden

*** Correspondence:**
Tobias Schmidt
tobias.schmidt@med.lu.se

1 Supplementary Figures

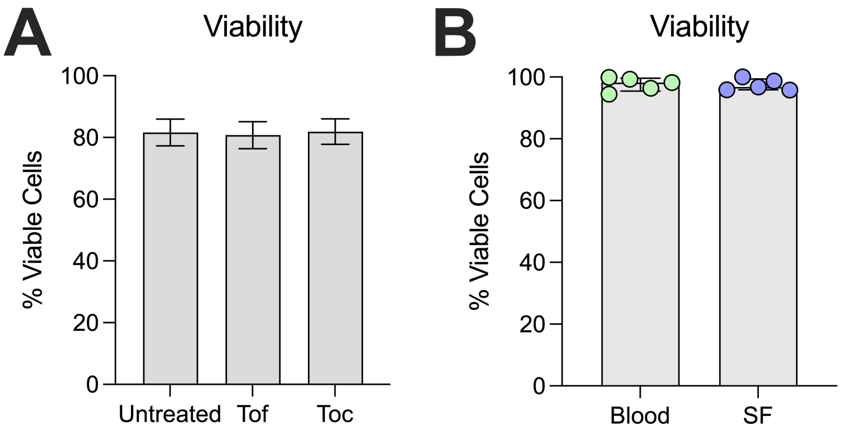

Supplementary Figure 1. Monocyte viability (**A**) Monocytes were pre-treated with tocilizumab (100ng/ml) or tofacitinib (1µM) before being treated with a pool of synovial fluid (SF) from 6 oJIA patients and polarized overnight. Viability was assessed using Annexin V / PI staining and flow cytometry. Viable cells were defined as Annexin V / PI negative. Each condition was performed in triplicate, and the bars represent mean ±SD of 3 different monocyte donors. (**B**) A subset of patients (n=5) was initially evaluated for differences in viability in blood vs synovial monocytes by staining using a live/dead dye. Monocytes were identified based on CD14 expression. Data is presented as median with interquartile range. *oJIA- oligoarticular juvenile idiopathic arthritis, PI- Propidium iodide, tof- Tofacitinib, toc- Tocilizumab, SF – Synovial fluid.*

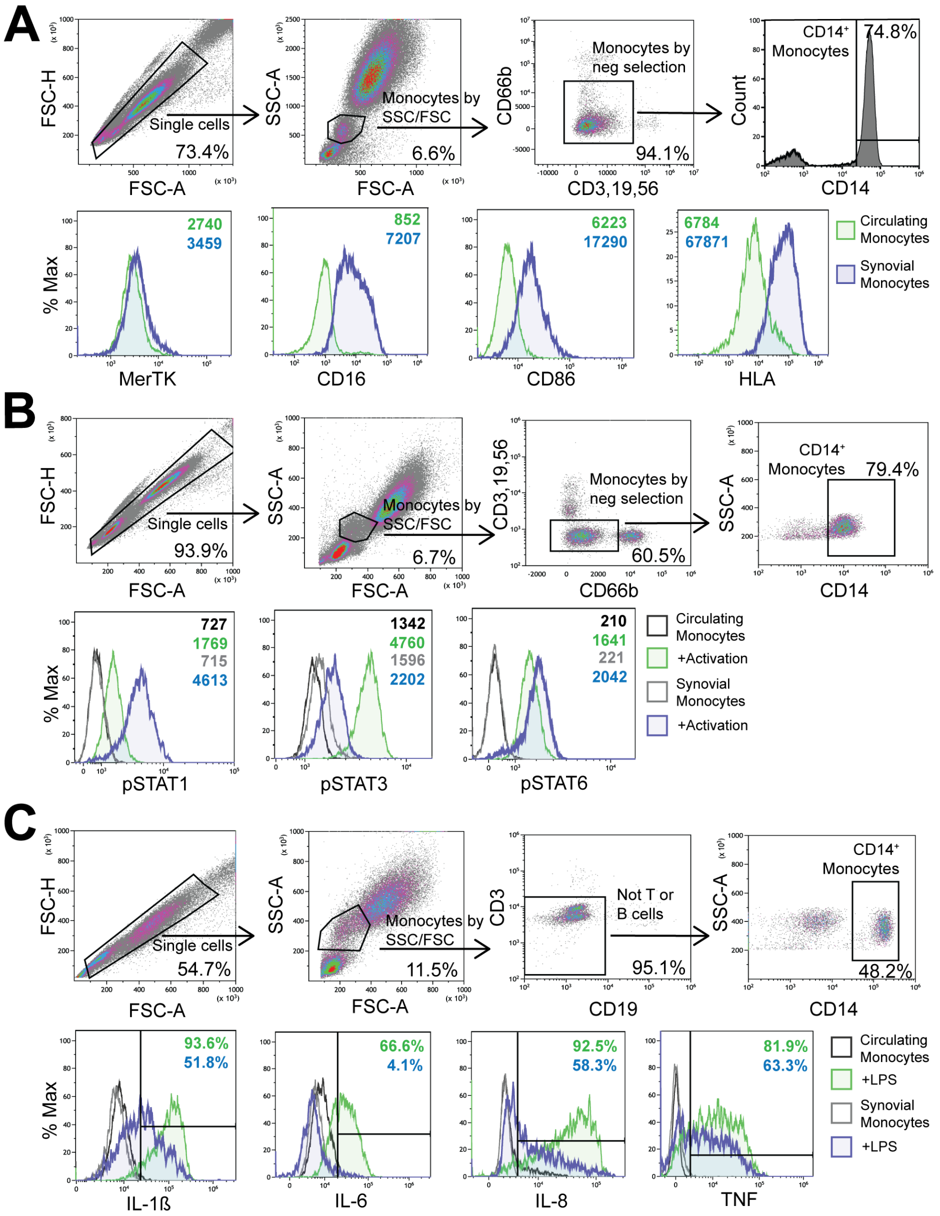

Supplementary Figure 2. Gating strategy used for monocytes in patients (**A**) Representative gating strategy used for the analysis of surface markers of monocytes in synovial fluid (SF) and blood. (**B**) Representative gating strategy used for the analysis of STAT phosphorylation following activation in SF and blood. (**C**) Representative gating strategy for the analysis of intracellular cytokine production following LPS activation in SF and blood. *LPS- Lipopolysaccharide, IL-Interleukin, STAT- Signal transducer and activator of transcription, MFI- Median fluorescence intensity, TNF- Tumor necrosis factor.*

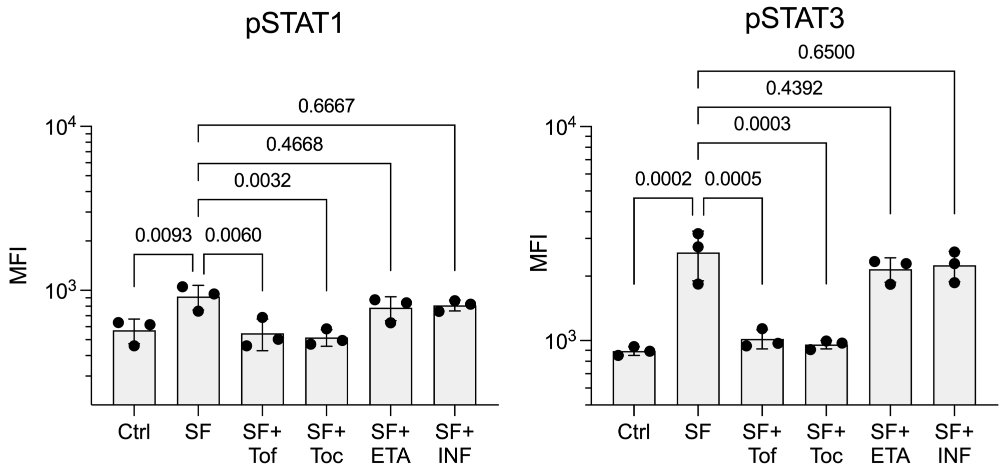

Supplementary Figure 3. Minimal effect of TNF inhibitors on the SF induced STAT phosphorylation Monocytes from n=3 donors were pre-treated with tofacitinib (1µM), tocilizumab, etanercept or infliximab (all 100ng/ml) for 25min, followed by activation with 20% SF (pooled from n=8 patients) for 10min. The control received no SF or drugs. The cells were subsequently fixated and stained for phosphorylated STAT1 and STAT3 and analysed by flow cytometry. Statistics were performed using one-way ANOVA. *SF- synovial fluid, IL- Interleukin, STAT- Signal transducer and activator of transcription, MFI- Median fluorescence intensity, Tof – Tofacitinib, Toc – Tocilizumab, ETA – Etanercept, INF – Infliximab.*

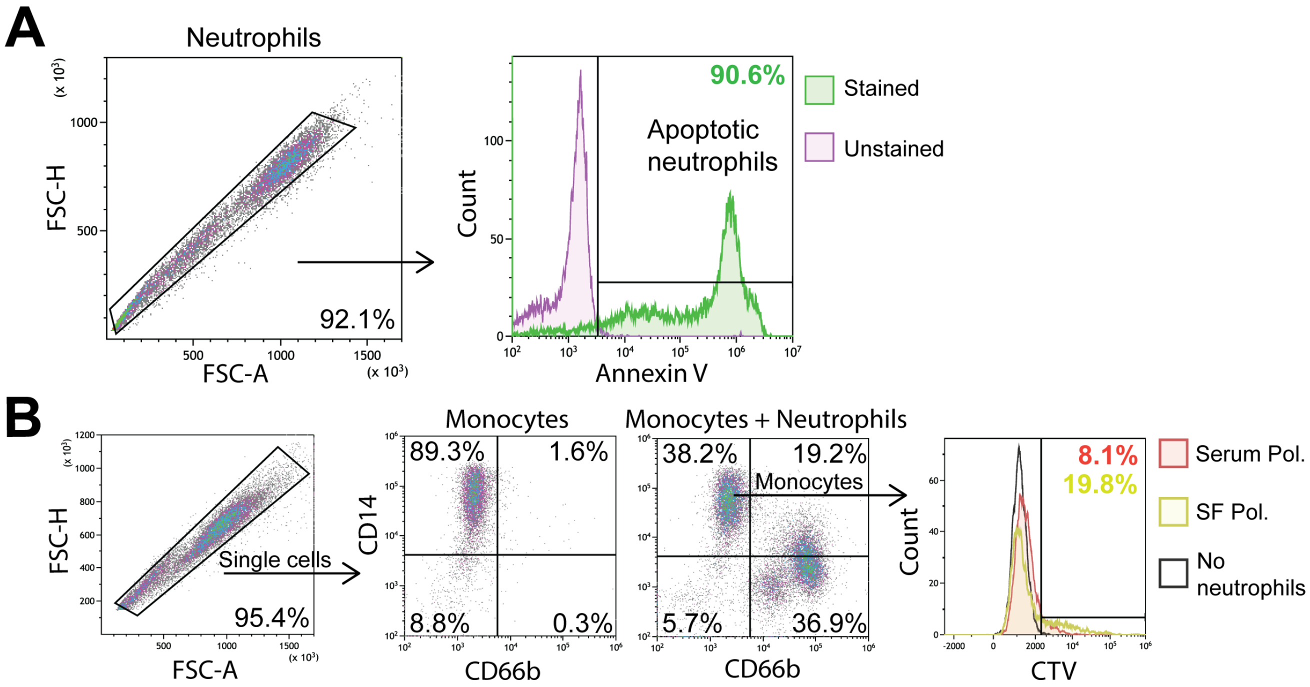

Supplementary Figure 4. Generation of apoptotic neutrophils and gating strategy for efferocytosis Neutrophils were isolated from healthy donors, stained with CTV, and incubated overnight in serum poor medium. (**A**) A representative experiment showing how apoptosis was confirmed for each experiment using Annexin V staining. (**B**) Representative gating strategy, showing serum and SF polarized monocytes which were gated as $CD14^{+}CD66b^{-}$ to exclude monocytes that bound, but did not internalize, apoptotic neutrophils. *CTV- Cell Trace Violet, SF- synovial fluid, Pol- Polarization.*

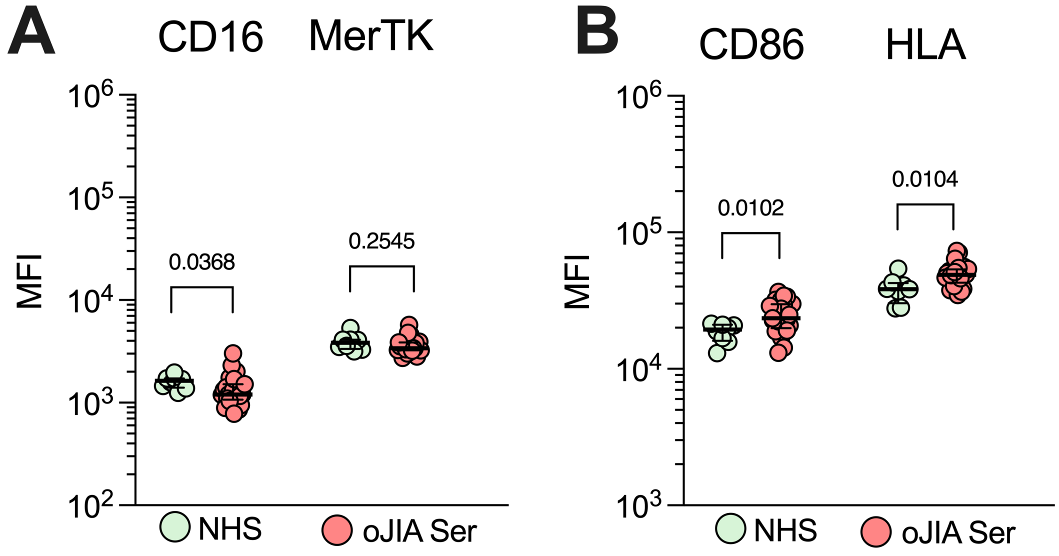

Supplementary Figure 5. Sera from oJIA patients do not induce a regulatory phenotype compared to normal human serum Monocytes were isolated from healthy controls and, as a control to patient serum (n=24), they were also polarized with pooled normal human serum (NHS, n=8) in each experiment. The monocytes were analysed by flow cytometry for surface expression of (**A**) CD16 and MerTK, as well as (**B**) CD86 and HLA. Data represents median with interquartile range. Statistics were performed using Mann-Whitney U test.

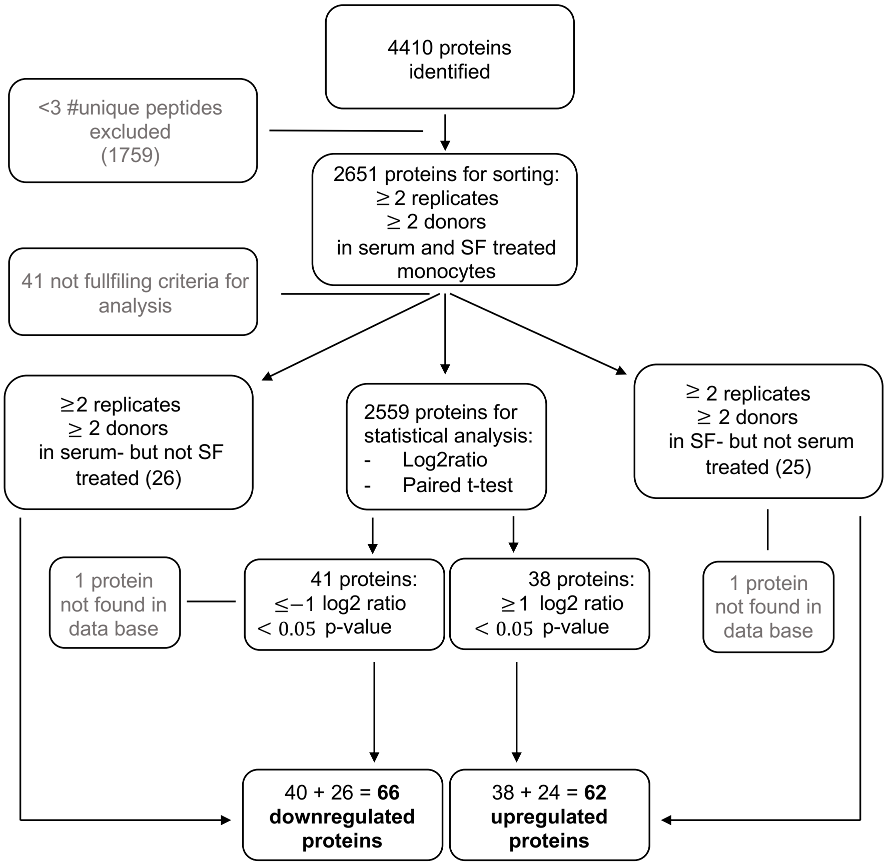

Supplementary Figure 6. Strategy to identify down- and upregulated proteins identified by mass spectrometry in serum vs synovial fluid polarized healthy monocytes Monocytes from n=3 healthy donors were polarized with a pool of serum or synovial fluid (SF) from patients with oJIA, and subsequently analysed by mass spectrometry. Proteins were next sorted and analysed, resulting in 66 downregulated proteins and 62 upregulated proteins, which were then used for gene ontology enrichment analysis of biological processes.

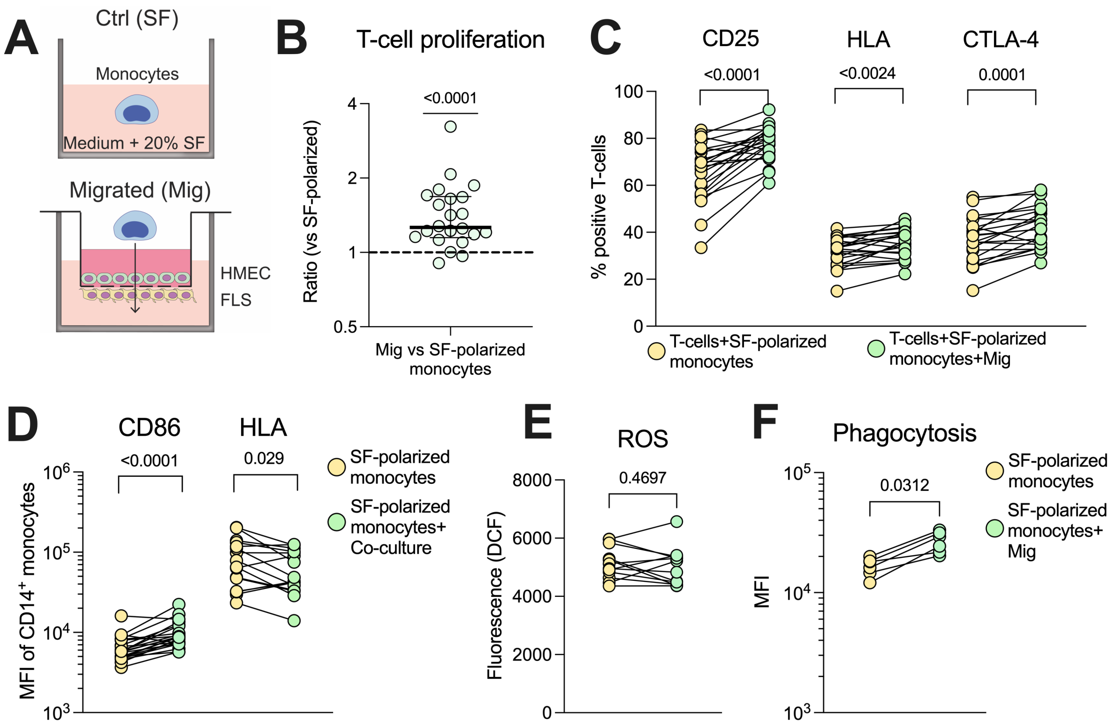

Supplementary Figure 7. Migration induces some of the features observed in the patients' monocyte phenotype (**A**) Experimental setup of the migration assay, in which monocytes were allowed to migrate for 3hrs followed by removal of the inserts and polarization overnight (**B**) Non-migrated control (SF) and migrated (Mig) monocytes were detached and co-cultured with CTV-stained CD3 activated T-cells (1:10 monocytes to T-cells) for 72hrs, followed by analysis of proliferation (displayed as ratio of percent proliferation between migrated vs non-migrated monocytes) and (**C**) expression of activation markers in T-cells (n=22). (**D**) Shows changes in surface expression of CD86 and HLA in non-migrated vs migrated monocytes. (**E**) Displays ROS production after 1hr incubation following H_2DCFDA staining (n=12) and (**F**) phagocytosis of opsonized FITC labelled beads for 30min (n=6). Wilcoxon matched pairs signed rank test. Lines at median. *HMEC- Human dermal microvascular endothelial cells, FLS- Fibroblast-like synoviocytes, ROS- Reactive oxygen species, MFI- Median fluorescence intensity, SF- Synovial fluid.*

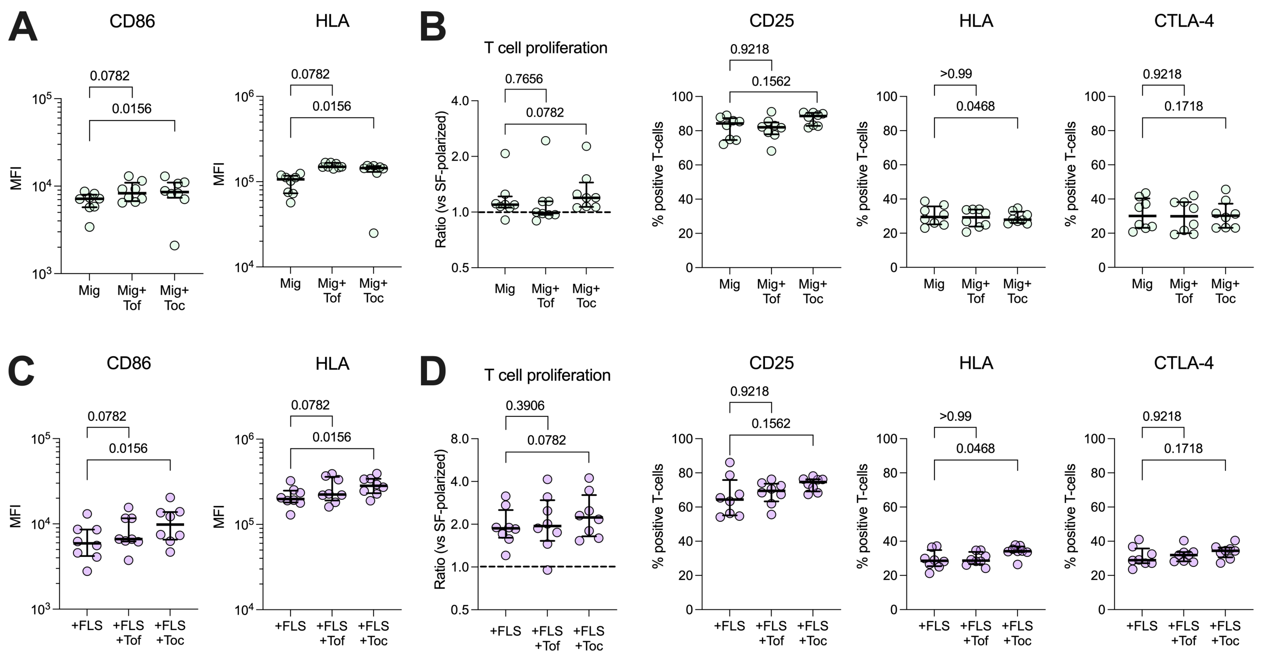

Supplementary Figure 8. Tofacitinib and tocilizumab do not inhibit the increased antigen presentation abilities by migrated or co-cultured monocytes (**A**) Monocytes were pre-treated with tofacitinib (1µM) or tocilizumab (100ng/ml) before migration for 3hrs, followed by polarization overnight. Next day, they were analysed for expression of CD86 and HLA, or (**B**) detached and incubated with healthy CD3 activated T cells for 72hrs, which were analysed for proliferation and surface marker expression. (**C**) Instead of migration, monocytes were polarized and co-cultured with fibroblast-like synoviocytes overnight, and analysed for CD86, HLA or (**D**) T-cell activation. Wilcoxon matched pairs signed rank test, n=8. Lines at median with interquartile range. *MFI- Median fluorescence intensity, SF- Synovial fluid, FLS- Fibroblast-like synoviocytes, Tof- Tofacitinib, Toc- Tocilizumab, Mig- Migrated monocytes.*

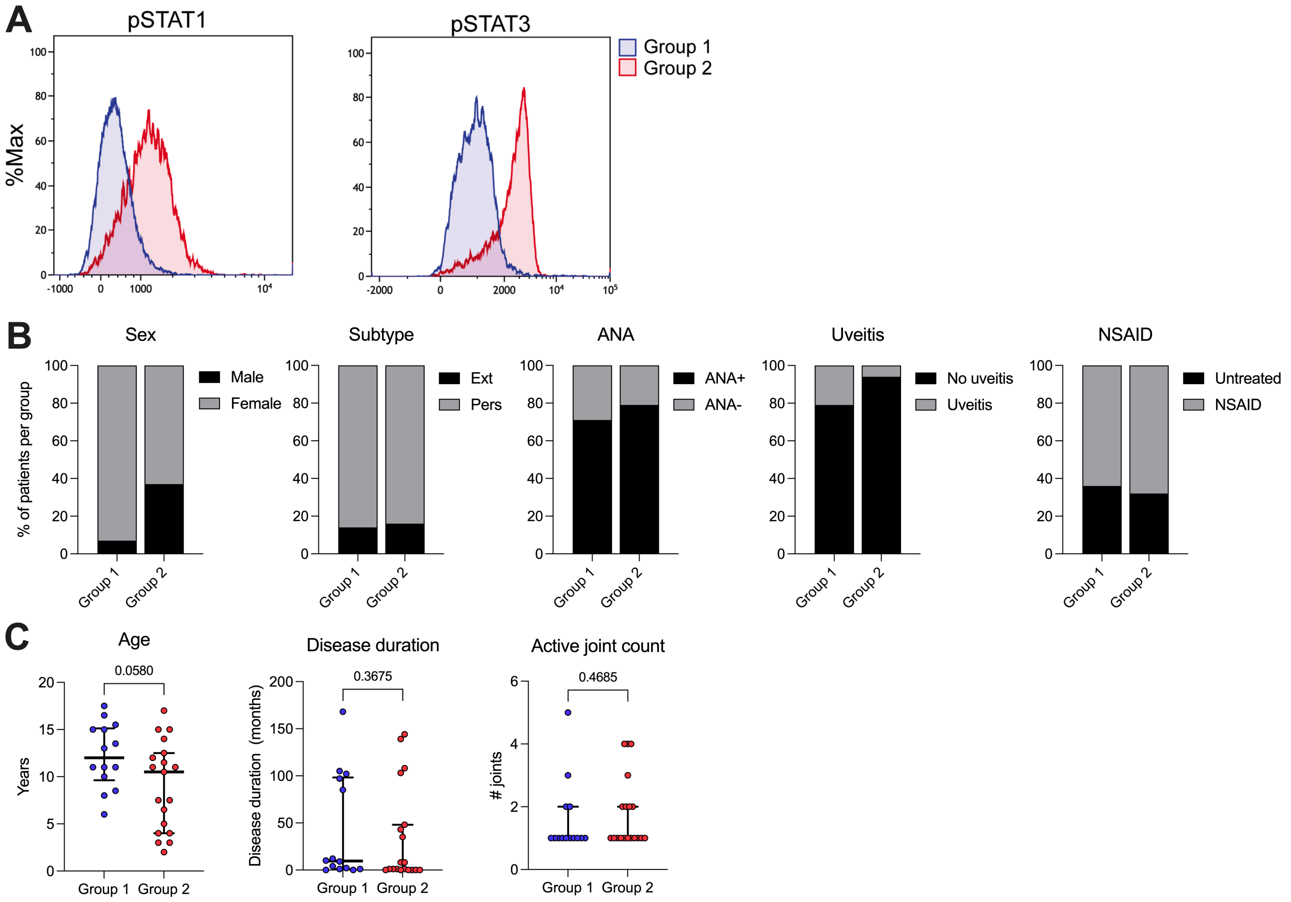

Supplementary Figure 9. Features of the two patient groups Patients were categorized by hierarchical clustering based on IL-6/JAK/STAT into group one (low IL-6/JAK/STAT involvement, n=14) and group two (high IL-6/JAK/STAT involvement, n=19).(**A**) Shows representative histograms of pSTAT1 and pSTAT3 expression in a patient from group 1 and a patient from group 2. **B-C** shows clinical characteristics in the two different groups (**B**) Shows percentage of the respective groups. (**C**) Displays the median with interquartile range. Statistics were performed with Mann-Whitney U test.
